# Resource-dependent biodiversity and potential multi-trophic interactions determine belowground functional trait stability

**DOI:** 10.1186/s40168-023-01539-5

**Published:** 2023-05-01

**Authors:** Lingyue Zhu, Yan Chen, Ruibo Sun, Jiabao Zhang, Lauren Hale, Kenneth Dumack, Stefan Geisen, Ye Deng, Yinghua Duan, Bo Zhu, Yan Li, Wenzhao Liu, Xiaoyue Wang, Bryan S. Griffiths, Michael Bonkowski, Jizhong Zhou, Bo Sun

**Affiliations:** 1 State Key Laboratory of Soil and Sustainable Agriculture, Institute of Soil Science, Nanjing, 210008 China; 2 University of Chinese Academy of Sciences, Beijing, 100049 China; 3 Anhui Province Key Laboratory of Farmland Ecological Conservation and Pollution Prevention, College of Resources and Environment, Anhui Agricultural University, Hefei, 230036 China; 4 Institute for Environmental Genomics, University of Oklahoma, Norman, OK 73019 USA; 5 United States Department of Agriculture, Agricultural Research Service (ARS), Washington, DC 20250 USA; 6 Terrestrial Ecology, Institute of Zoology, University of Cologne, 50674 Cologne, Germany; 7 Laboratory of Nematology, Wageningen University & Research, Wageningen, 6708 PB The Netherlands; 8 Department of Terrestrial Ecology, Netherlands Institute of Ecology (NIOO-KNAW), Wageningen, 6700AB The Netherlands; 9 CAS Key Laboratory for Environmental Biotechnology, Research Center for Eco-Environmental Sciences, Chinese Academy of Sciences, Beijing, China; 10 College of Resources and Environment, University of Chinese Academy of Sciences, Beijing, 100081 China; 11 Institute of Agricultural Resources and Regional Planning, Chinese Academy of Agricultural Sciences, Beijing, 100081 China; 12 Institute of Mountain Hazards and Environment, Chinese Academy of Sciences, Chengdu, 610041 China; 13 Xinjiang Institute of Ecology and Geography, Chinese Academy of Sciences, Urumqi, 830011 China; 14 Institute of Soil and Water Conservation, Chine, Academy of Sciences and Ministry of Water Resources , Yangling, 712100 China; 15 SRUC, Crop and Soil System Research Group, West Mains Road, Edinburgh, EH93JG UK

**Keywords:** Soil biodiversity, Within trophic interactions, Cross-trophic interactions, Agroecosystem, Ecosystem functioning

## Abstract

**Background:**

For achieving long-term sustainability of intensive agricultural practices, it is pivotal to understand belowground functional stability as belowground organisms play essential roles in soil biogeochemical cycling. It is commonly believed that resource availability is critical for controlling the soil biodiversity and belowground organism interactions that ultimately lead to the stabilization or collapse of terrestrial ecosystem functions, but evidence to support this belief is still limited. Here, we leveraged field experiments from the Chinese National Ecosystem Research Network (CERN) and two microcosm experiments mimicking high and low resource conditions to explore how resource availability mediates soil biodiversity and potential multi-trophic interactions to control functional trait stability.

**Results:**

We found that agricultural practice-induced higher resource availability increased potential cross-trophic interactions over 316% in fields, which in turn had a greater effect on functional trait stability, while low resource availability made the stability more dependent on the potential within trophic interactions and soil biodiversity. This large-scale pattern was confirmed by fine-scale microcosm systems, showing that microcosms with sufficient nutrient supply increase the proportion of potential cross-trophic interactions, which were positively associated with functional stability. Resource-driven belowground biodiversity and multi-trophic interactions ultimately feedback to the stability of plant biomass.

**Conclusions:**

Our results indicated the importance of potential multi-trophic interactions in supporting belowground functional trait stability, especially when nutrients are sufficient, and also suggested the ecological benefits of fertilization programs in modern agricultural intensification.

Video Abstract

**Supplementary Information:**

The online version contains supplementary material available at 10.1186/s40168-023-01539-5.

## Background

Belowground organisms contribute to ecosystem functioning through their effects on physical properties and biological processes [1]. Intensive land use with resource inputs drives changes in the soil biotic community structure, which ultimately impacts the maintenance of soil functionality and crop productivity [2–4]. Therefore, quantifying the effect of resource levels on soil biota is a step toward maintaining the sustainability of intensive agricultural ecosystems. Different from plant communities, in which heterogeneous resource acquisition stabilize crop production [5], the belowground species for nutrient acquisition to influence the biotic community are far more multifaceted. Millions of bacteria, fungi, protists, and nematodes, among others, make up soil communities with multiple trophic levels and form complex ecological interaction webs [6, 7]. These multi-trophic groups in manipulated experiments [8, 9], and at global scale [1, 10–12], have been observed to regulate the capacity of ecosystems to provide multiple services. Because the functional effects of any trophic group may depend on the abundance and diversity of others [13], resource-driven environmental selection has different impacts on the biodiversity involved in various trophic groups. For example, resource deficiency not only provides niche for oligotrophic microbial species growth by limiting opportunists that are poorly adapted to local environment [14] but also has been found to shift high trophic-level species diversity [15–18]. Theoretically, this may lead to the functional effects of multi-trophic groups complementing or opposing each other. However, whether and how resource-driven alteration of biodiversity further changes the soil functional stability has not been assessed.

Besides soil biodiversity, the large numbers of individual species belowground also establish a myriad of positive and negative, direct and indirect interactions to stabilize ecosystem functions [7, 19, 20]. Heterogeneous resource utilization by decomposers (so-called bottom-up forces) and higher trophic species (top-down forces) profoundly affect the assembly of multi-trophic ecological webs [13, 21, 22] through frequent interactions among the within trophic groups (soil organisms occupying the same levels in the food chain) and cross-trophic groups (soil organisms occupying the different levels in the food chain) [23]. Under laboratory conditions, high nutrient concentrations have been shown to increase the number of negative interactions between bacterial species and result in the loss of species richness and decreased stability of the microbial community [24]. Conversely, in the intensive cropping fields, soil with periodic input of resources (such as fertilizer and organic matter inputs), compared with no resource application, enhanced microbial functional diversity [25, 26]. Besides within trophic interactions, the intensity of grazing between bacteria and protists can be influenced by the supply of carbon and phosphorus resources [27, 28]. However, most previous studies [8, 24] typically focused on the resource regulation of single trophic level, neglecting the fact that shifts in nutrient conditions have cyclic impacts on the biodiversity and multi-functionality across multiple trophic levels by both top-down and bottom-up forces [29]. Therefore, exploration of resource-driven, potential multi-trophic interactions, and their effects on functional stability, will provide a clearer overall perspective for the role played by the complex community of organisms in driving ecosystem services.

Empirical community ecology holds that taxonomically different organisms can contain similar functional traits which ensure that the extinction of individual species, due to external disturbances, does not cause a collapse of the entire functional system [30]. As a result, the degree of functional redundancy, to a certain extent, can be used to estimate the functional stability in a given ecosystem [31]. In this study, we used functional trait stability, the redundancy of individual biochemical functional genotypic trait (R) [32] and the inverse of the coefficient of variation of R [33] of given multiple functional genes from a functional gene-array-based high-throughput technology [34], to evaluate the magnitude of soil potential functional stability in intensive agroecosystems. We hypothesized that long-term impacts of anthropogenic resource change on belowground functional trait stability strongly depend on how such drivers influence biodiversity and potential multi-trophic interactions. We collected soil samples (including high and low resource categories from 4 treatments) at 5 typical agro-ecological experimental stations that have been continuously cultivated for over 25 years from the Chinese National Ecosystem Research Network (CERN). Using these multi-site field investigations and the two controlled microcosm experiments, we attempt to answer (i) does resource availability mediate the effects of soil biodiversity and potential multi-trophic interactions on functional trait stability? and (ii) if so, how does the relative importance of biodiversity and potential multi-trophic interactions change for stabilizing functional traits at different resource levels?

## Methods

### Field site description, sampling, and resource level classification

Samples with different resource availability were collected from 4 treatments of 5 agroecosystem field stations located in Fengqiu (FQ), Qiyang (QY), Changwu (CW), Yanting (YT), and Fukang (FK) (Figure S1). These stations belong to the Chinese National Ecosystem Research Network (CERN, http://www.cern.ac.cn), and experiments were established in 1980. Data of mean annual temperature and mean annual precipitation in each site were obtained from the website of Weather China (http://www.weather.com.cn). The 4 treatments of different resource applications: (I) C, no resource application; (II) NK, chemical nitrogen (N) and potassium (K) applied but without phosphorus (P); (III) NPK, chemical N, P and K applied; (IV) NPKM, application of chemical NPK plus organic manure. Details of fertilizer amount and crop planting (including soybean, maize, and wheat) are described in Table S1. These treatments were conducted as randomly distributed triplicate plots in each field site. In total, 60 soil samples (5 sites × 4 treatments × 3 replicates) were collected after spring crop harvest in 2015. Soil chemical properties were measured using standard methods (Supplementary Methods) and listed in Table S2.

To obtain a quantitative index of soil resource availability for each sample, the resource-relevant soil properties (including soil organic carbon (SOC), total nitrogen (TN), total phosphorus (TP), total potassium (TK), the ratio of ammonium nitrogen and nitrate nitrogen to total nitrogen (NH_4_^+^-N+NO_3_^-^-N):TN, the ratio of available phosphorus to total phosphorus (AP:TP) and the ratio of available potassium to total potassium (AK:TK)) were individually standardized using the following equation.

1 $$S_{\mathrm{RP}}=\frac{RP-RP_{min}}{RP_{max}-RP_{min}}$$

where RP is the resource-relevant soil properties (SOC, TN, TP, TK, (NH_4_^+^-N + NO_3_^−^-N):TN, AP:TP and AK:TK) of samples, ${RP}_{\mathrm{min}}$ is minimum RP value, and ${RP}_{\mathrm{max}}$ is maximum RP value across all samples.

**Fig. 1:**
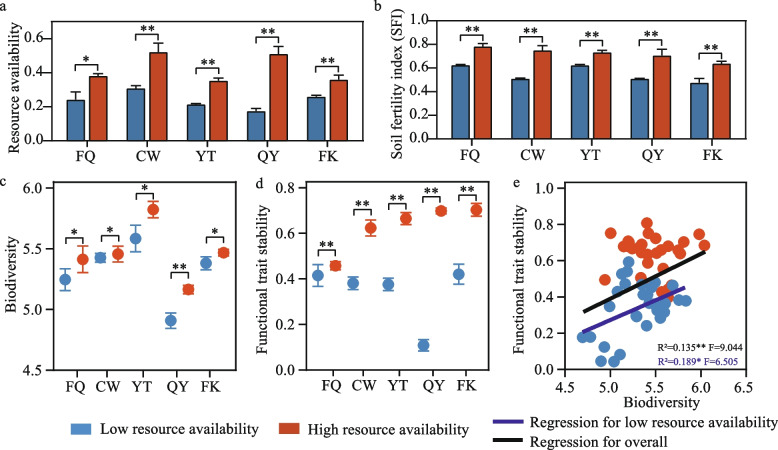
The composite resource availability indicator (CRAI), soil fertility index (SFI), biodiversity, functional trait stability, and their relationships between low and high resource availability environments. **a** The differences of CRAI between low and high resource category at each sampling site. **b** The differences of SFI between low and high resource environments at each sampling site. **c**, **d** Comparisons of biodiversity and functional stability at each sampling site. Asterisks denote significant differences between metrics for low and high resource availability soils within each site (*n* = 6) (Wilcoxon rank-sum test, *, *P* < 0.05; **, *P* < 0.01). **e** The linear relationships between belowground biodiversity and functional trait stability. The blue and black solid lines represent the significant relationships in low-resource sites and all sampling sites, respectively. No significant relationship was found in high resource availability soils

The standardized samples were then averaged across resource-relevant soil properties to represent resource availability. We then classified samples of each site into low and high resource categories according to resource availability (Fig. 1a).

To verify the reliability of resource classification, the soil fertility index (SFI) of each sample among sites was evaluated using the equation of Integrated Quality Index [35, 36] as follows:

2 $$\mathrm{IQI}={\sum }_{i=1}^{n}{W}_{i}\times {S}_{i}$$

where ${W}_{i}$ is the weight of indicator* i* in farmland sites containing *n* common chemical indicators (concentration of organic matter, AP, AK, and soil pH) from National Cultivated Land Quality Grade [37]. ${S}_{i}$ is the score of indicator *i* calculated by the model of score function from National Cultivated Land Quality Grade supported in Table S3.

### Soil biodiversity measures

High-quality 1000 ng soil DNA (260/280 and 260/230 ratios ≥ 1.8, NanoDrop ND-1000 spectrophotometer, NanoDrop Technologies, Delaware, USA) were extracted from each sample (Supplementary methods) and used for molecular analysis. Complete documentation of the high-throughput sequencing is also provided in Supplementary methods and specific primer information for bacteria, fungi, protists, and nematodes are presented in Table S4. The raw read counts (Dataset S1 and S2) were rarefied and then transformed to relative abundances (separately for bacteria, fungi, protists, and nematodes) and merged into a new integrated operational taxonomic units (OTU) table [38]. We used this integrated OTU table for the subsequent analyses.

In this study, the integrated Shannon index (including OTUs of bacteria, fungi, protists, and nematodes) calculated from the integrated OTU table was used to determine the biodiversity. To prove the rationality of the calculated biodiversity, the diversity (richness, Shannon, and evenness) of soil bacteria, fungi, protists, and nematodes were individually standardized using the following Eq. (3) from previous publications [39, 40]:

3 $$Bio_{ref}\left(\mathrm{diversity}\right)=\frac{RD-RD_{min}}{RD_{max}-RD_{min}}$$

where RD is the raw diversity (richness, Shannon, and evenness) of the sample, ${\mathrm{RD}}_{\mathrm{min}}$ is minimum diversity value, and ${\mathrm{RD}}_{\mathrm{max}}$ is maximum diversity value across all samples.

The standardized samples were then averaged across organism groups to represent Bio_ref_ (diversity). As we expected, the biodiversity (integrated Shannon) calculated from the integrated OTU table was highly correlated with that of Bio_ref_ (richness), Bio_ref_ (Shannon), and Bio_ref_ (evenness) both in low and high resource groups (Figure S2) and the diversity (richness, Shannon, and evenness) of each group of soil organisms (Figure S3), indicating integrated Shannon can be used to characterize the biodiversity of the overall community.

### Soil functional trait stability measures

We used DNA-based microarray GeoChip 5.0 to assess soil functional traits based on genotypic characteristics (Supplementary methods) [34]. Functional trait stability was calculated by the redundancy of individual biochemical functional traits (R) [32] (Dataset S3) and the inverse of the coefficient of variation of R (1/CV) [33]. The 1/CV is calculated by the ratio of the mean (μ) to the standard deviation (σ) of R in each sample [41] as:

4 $$\frac{1}{CV}=\frac{u}{\upsigma }=\frac{\overline{F} }{\sqrt{\frac{\sum_{i=1}^{n}{({F}_{i}-\overline{F })}^{2}}{n}}}$$

where $\overline{\mathrm{F} }$ being the mean of the frequency of all functional traits in the sample, ${\mathrm{F}}_{\mathrm{i}}$ is the frequency of the ith functional trait, and n is the number of functional traits in the sample.

The standardization of R and 1/CV refers to the calculation method of Bio_ref_ (diversity) in the manuscript and then takes the average value of R and 1/CV of standardized samples to calculate functional trait stability according to:

5 $$\mathrm{Functional\,trait\,stability}=\frac{1}{2}\times (s(R)+s(\frac{1}{CV}))$$

where $\mathrm{s}(\mathrm{R})$ and $\mathrm{s}(\frac{1}{\mathrm{CV}})$ denote standardized R and 1/CV, respectively.

### Microbial network construction and potential multi-trophic interactions measures

We used the Spearman correlation matrix (|*r*|> 0.6, *P* < 0.05) to construct co-occurrence networks through the *WGCNA* package [42]. This promising approach is widely used to discover the co-occurrence correlation between OTUs [43–46]. Nodes in all networks represent OTUs and the links that connect these nodes represent correlations between OTUs [47]. We adjusted all P-values for multiple testing using the Benjamini and Hochberg false discovery rate (FDR) controlling procedure by “multtest” R (version 3.6.1) package [48]. The cutoff of FDR-adjusted *P*-values was 0.05. Network properties were calculated with the “igraph” package. Correlation networks were visualized using Gephi (version 0.9.1) and Cytoscape (version 3.7.2) software.

**Fig. 2:**
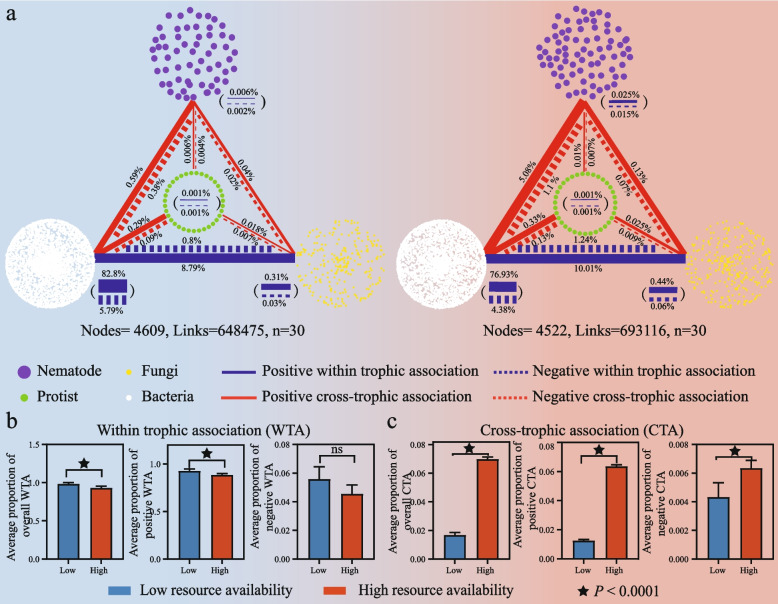
The construction of integrated co-occurrence networks of soil organisms (including bacteria, fungi, protists, and nematodes) and proportions of potential multi-trophic interactions types in low and high available resource environments. **a** Visualization of integrated co-occurrence networks across low (left) and high (right) resource availability environments based on Spearman correlation matrix (|*r*|> 0.6, *P* < 0.05). Each node represents OTUs. These nodes were clustered according to the category of kingdoms and marked with purple (nematode), green (protist), yellow (fungi), and white (bacteria) colors. Each link between the same and different kingdoms represents a significant pairwise association between them. The color of links was used to distinguish trophic levels between nodes. Blue links represent within trophic associations (potential within trophic interactions), while red links represent cross-trophic association (potential cross-trophic interactions). The shape of links was used to distinguish the impact of associations. The solid and dashed lines represent positive and negative associations between nodes, respectively. The thickness of links represents the proportion of a certain type of association in total associations. **b**, **c** Average proportion of within trophic (WTA) and cross-trophic associations (CTA) in low and high available resource. Asterisks denote significant differences between metrics for low and high resource availability soils (*n* = 30) (Wilcoxon rank-sum test, asterisk, *P* < 0.0001; ns, not significant)

Co-occurrence networks of soil organisms were constructed for low and high resource availability independently based on the integrated OTU table (including bacteria, fungi, protists, and nematodes datasets). The OTUs were filtered by setting 20 as the minimum occurrence across 30 of low and high resource samples, respectively. The proportions of multiple association types (positive/negative within trophic and positive/negative cross-trophic associations) and network properties were calculated by extracting sub-networks for each soil sample from the integrated networks [49] (Fig. 2a). Then, to determine the consistency of the resource-driven association patterns found in integrated networks, we re-constructed 10 site-dependent co-occurrence networks (2 resource levels × 5 sites) (Figure S4).

To distinguish the potential multi-trophic interactions in the co-occurrence networks, the connections between the same trophic level were classified as within trophic associations and connections between different trophic levels were classified as cross-trophic associations. The two classifications represent potential within and cross-trophic interactions, respectively [23, 50]. In total, 20 types of potential species interactions were divided into four categories: positive within trophic, negative within trophic, positive cross-trophic, and negative cross-trophic interactions (Table S5). We considered bacteria and fungi as the basal trophic level and protist and nematode as the high trophic level in this study.

### Microcosm study

To determine whether the pattern of functional trait stability regulation found in the field is consistent at the microdomain scale, we designed two microcosmic experiments. These were conducted in liquid culture, independent from the field experiments presented above, enabling us to test the results from the field experiments. Following the protocol outlined by Bonkowski et al. [51], we used full-strength wheat grass medium (WG) and 1/100 diluted WG to imitate high- and low-resource liquid incubation systems.

For experiment 1, soil biota from 20 g fresh soil of C and NPKM treatments (representing typical original low and high resource environments) of the Yanting Ecological Experimental Station (Table S1) were gently shaken and suspended in 100 mL phosphate buffer (PBS). Then, 500 μL soil suspension containing soil biota was added to 5 mL WG and 1/100 diluted WG liquid medium, respectively, and incubated at 26 °C on a rotating shaker at 120 rpm in the dark. After 4 days, 500 μL of culture was transferred to fresh medium for subculturing, and the remaining culture was collected for microbial carbon metabolic profiles using Biolog EcoPlates. Subculturing was performed 3 times. After 12 days of incubation, the subcultures were collected for DNA extraction and high-throughput sequencing of organisms (including bacteria, fungi, protists, and nematodes). To assess the functional stability (temporal stability of functional traits based on microbial carbon metabolism) of the microcosm, Biolog EcoPlates (Biolog® Inc., CA, USA) was used to measure the average well color development (AWCD) at the end of each subculturing by tracking its temporal dynamics (Figure S5, Database 4). It is a simple and high-sensitivity method to determine the overall functional traits of microbiota [52–54]. By measuring the invariability of functional traits at different time intervals, we quantified functional stability as the inverse of the coefficient of variation of AWCD (μ/σ) [55, 56], which is the ratio of the mean to the standard deviation of the AWCD of each subculturing over time, and the formula is as follows:

6 $$\mathrm{Functional\,stability}=\frac{u}{\upsigma }=\frac{\overline{A} }{\sqrt{\frac{\sum_{i=1}^{n}{({A}_{i}-\overline{A })}^{2}}{n}}}$$

where $\overline{\mathrm{A} }$ being the mean of the AWCD in the sample, ${\mathrm{A}}_{\mathrm{i}}$ is the AWCD of the ith subculturing, and n is the number of subculturing in the sample.

The Biolog EcoPlates consisted of 96-well microplates containing 31 different carbon sources plus three replicate blank wells. Microbial extraction and incubation in the Biolog EcoPlates followed the protocol outlined in Chen et al. [57].

For experiment 2, we used heat-killed and selectively filtered soil suspension to create different trophic micro-systems for culturing the model plant *Arabidopsis thaliana*. Soil suspension of field NPKM was prepared as mentioned above (experiment 1). Then 100 mL suspension were divided into 3 aliquots. One (suspension i) was heat-killed through 120 °C for 20 min. Another (suspension ii) was filtered through 1-µm sterile filter to remove large size body species including fungi, protists, and nematodes. The third (suspension iii) was untreated to keep all species alive. Then the three suspensions were respectively mixed with WG and 1/100 WG in the volume ratio of 1:10 to make 6 types of medium (i + WG, ii + WG, iii + WG, i + 1/100 WG, ii + 1/100 WG, iii + 1/100 WG) for culturing Arabidopsis thaliana seedlings. The culture systems were divided into 3 categories according to soil suspensions: system I, the axenic culture including i + WG and i + 1/100 WG; system II, within trophic-level culture including ii + WG and ii + 1/100 WG; system III, multi-trophic-level culture including iii + WG and iii + 1/100 WG.

Seeds of wild-type *Arabidopsis thaliana* were surface-sterilized using 70% ethanol and 1.5% NaClO [38]. Individual seeds were sown onto the surface of 1/2 Murashige and Skoog (MS) solid medium (Sigma-Aldrich M5519, pH 5.7). The medium were incubated for 2 days at 4 °C in the dark and then transferred to a light incubator (14 h light/10 h dark, humidity 60%, temperature 20 °C) for germination [58]. After 10 days, seedlings with consistent size were transferred to a cell culture plate (flat bottom with lid). Four seedlings were placed together into each well (diameter = 2.4 cm; height = 1.8 cm) with degreasing cotton at the bottom, and then injecting 3300 µL of culture medium into the well. To simultaneously meet the development of plant *Arabidopsis thaliana* and microorganisms [59, 60], we set the temperature at 20℃ as ambient, temperatures reduced 5 ℃ below ambient as temperature stress [61, 62]. Each well of the micro-system represented a replicate. For each medium, 24 replicates were conducted, with twelve replicates incubated at 15 ℃, and another twelve replicates were incubated at 20 ℃. Other culture conditions (including light and humidity) were the same as for seed germination. In total, 576 seedlings (4 plants × 24 wells × 6 culture mediums) were transferred into 144 wells for the determination of plant growth stability in different trophic-level systems. Controls with water addition instead of culture medium were conducted in an identical manner. For each culture well, the medium was replaced with fresh aliquots at 4-day intervals. After 8 days of incubation, seedlings in each well (*n* = 4) were selected for fresh biomass detection. The stability of plant biomass (Database 5) in different trophic-level micro-systems was calculated by the biomass resistance under low temperature stress as described by Orwin and Wardle [63]:

7 $$\mathrm{Resistance}=1-(\frac{2\left|D0\right|}{C0+\left|D0\right|})$$

where D0 is the difference between the samples cultured at 20 ℃ (C0) and soil subjected to temperature stress (samples cultured at 15 ℃) at the end of the disturbance.

### Statistical analysis

Wilcoxon rank-sum test was used to assess the differences of soil quality index, biodiversity, functional stability, and soil biotic and abiotic characteristics between low and high resources in each site and one-way ANOVA was performed to assess the differences in plant biomass and resistance among different treatments using Tukey’s honest significant difference (HSD) tests (*P* < 0.05) in SPSS 20.0 software (SPSS, Chicago, IL, USA). The richness (Chao1 index), Shannon, and evenness indices were calculated with QIIME (Version 1.7.0). Random forest modeling was used to quantitatively assess the important predictors of functional trait stability involving soil characteristics and climatic factors. The analyses were conducted using the RandomForest package in R (version 3.6.1), and the significance of the model and predictor was determined using the rfUtilities and rfPermute packages [64, 65].

We used the Partial Least Squares Structural Equation Modeling (PLS-SEM) to estimate the driving factors directly and indirectly affect functional trait stability. PLS-SEM is more suitable for the estimation of very complex models with many latent variables and the analysis of less strict assumptions about the distribution of samples and error terms [66]. The PLS path modeling method was developed by Wold [67] and the PLS algorithm is essentially a sequence of regressions in terms of weight vectors [68]. The weight vectors obtained at convergence satisfy fixed point equations [69]. All PLS-SEM analyses were conducted using the Smart PLS 3.0 software (SmartPLS GmbH, Boenningstedt, Germany) [70]. To fit our model, we examined the fitting index of the model (Cronbach’s alpha > 0.7, composite reliability > 0.6, average variance extracted (AVE) > 0.5, path coefficients (*P* < 0.05)) (Table S6). The Goodness-of-Fit (GoF) index was established to evaluate the overall fitness of the model [71]. According to the GoF thresholds of 0.1, 0.25, and 0.36, the overall model fit was appropriately divided into weak, medium, and strong [72].

## Results

### Resource status affects the relationship between biodiversity and functional trait stability

To determine whether resource availability is important in controlling soil biodiversity, potential species interaction, and functional trait stability, we classified soil samples according to the resource availability in each site. As we expected, site samples in the high resource category showed greater resource availability (1.4–2.9 folds) than those in the low resource category (Fig. 1a, *P* < 0.05, Wilcoxon rank-sum test). We then used the soil fertility index (SFI, a common index for the evaluation of soil resource quality [73]) to support the rationality of the resource classifications (Table S3). The SFI of the high resource category ranged from 1.2 to 1.5 times higher than the low resource category among sites (Fig. 1b, *P* < 0.01, Wilcoxon rank-sum test), suggesting our classification represents actual nutrient availability.

In soil samples across agroecosystems, we found that low resource availability resulted in an average of 2.5 and 152% reduction in belowground biodiversity and functional trait stability (Fig. 1c,d, *P* < 0.05, Wilcoxon rank-sum test). Moreover, resource-driven average variability of functional trait stability (*P* < 0.01) was 50.43% greater than that of biodiversity (*P* < 0.05). After evaluating the relationship between biodiversity and functional trait stability, we found that soil biodiversity, in low resource availability samples only, exhibited strong linear correlations with functional trait stability (Fig. 1e, R^2^ = 0.189, F = 6.505 and *P* < 0.05). Once resource availability was sufficient, no significant relationship was observed (Fig. 1e, *P* > 0.05). Diversity of single groups of organisms (bacteria, fungi, protists, and nematodes) also yielded no significant relationships with functional trait stability under high resource conditions (Figure S6). However, if all samples were assessed as a single group, a significant positive relationship appeared again (Fig. 1e, R^2^ = 0.135, F = 9.044, and *P* < 0.01).

### Resource availability mediates soil potential multi-trophic interactions

To compare resource-driven potential species interaction differences, we constructed integrated co-occurrence networks (including bacteria, fungi, protists, and nematodes) of both low and high resource soils. In total, 648,475 and 693,116 associations (links) among 4609 and 4522 OTUs were captured in the low and high resource co-occurrence networks, respectively. A greater number of smaller microorganism nodes (bacteria and fungi) and their associated links were found than higher trophic nodes (protists and nematodes) and relevant links (Fig. 2a and Figure S4a). Then, we classified those links into within trophic (WTA) and cross-trophic (CTA) associations by judging whether the two nodes connected by links belonged to different trophic levels (Fig. 2b,c and Table S7). Obviously, the average proportion of overall WTA in the low resource network was higher than that in the corresponding high resource network (Fig. 2b, *P*< 0.01). This is attributed to the increase of positive WTA across low and high resource conditions (Fig. 2b and Figure S4b). By contrast, the increasing proportion of positive and negative cross-trophic associations made the average overall CTA across high resource networks 316% higher than those across low resource network (Fig. 2c). To further verify the consistency of resource-driven allocation of potential interactions between multi-trophic levels, we also constructed 10 site-dependent co-occurrence networks with high- and low-resource levels. These generated networks captured 6694–19,955 associations among 891–4117 OTUs (Figure. S4a, Table S7). Similarly, the variances of average WTA and CTA of site-dependent networks were consistent with integrated co-occurrence networks (Figure. S4b-c and Figure S7).

### Effects of potential multi-trophic interactions on functional trait stability

**Fig. 3:**
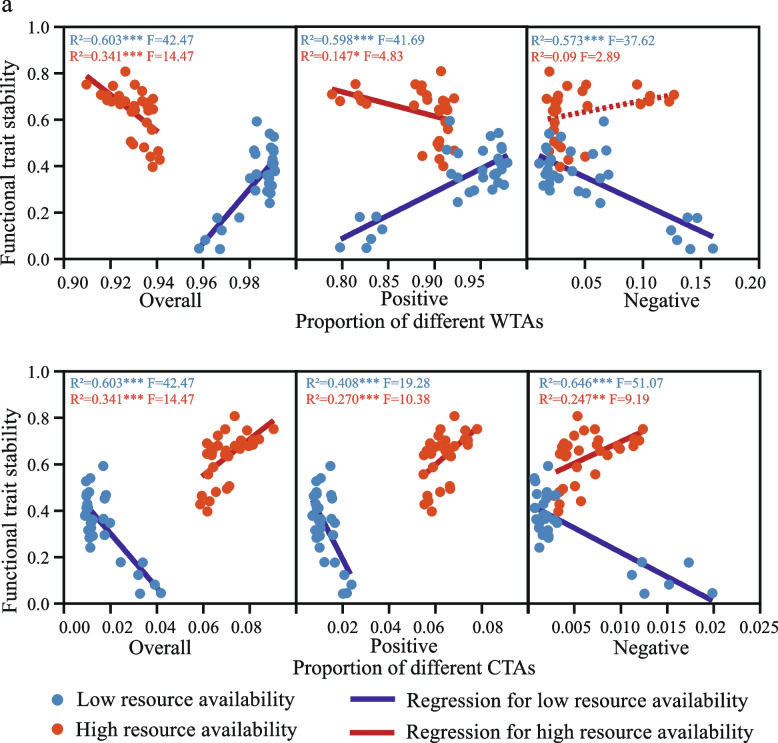
Types of trophic association effects on soil functional trait stability across low and high resource available environments. Blue and red dots represent samples from low and high resource treatments, respectively. Solid and dashed lines represent the significant and non-significant linear relationships in low and high resource environments, respectively. WTA, within trophic association; CTA, cross-trophic association. *, *P* < 0.05; **, *P* < 0.01; ***, *P* < 0.001

Using the common linear regression model, we found the different relations between potential multi-trophic interactions and functional trait stability (Fig. 3). Surprisingly, each type of association at low and high resource levels showed an opposite relationship with functional trait stability (R^2^ = 0.147–0.646; *P* < 0.05), although an individual linear relationship between negative WTA and functional trait stability was not significant (R^2^ = 0.09, *P* > 0.05) under high resource. That is, if the relation showed positive at low resource, then it turns to be negative at high resource, and vice versa. Another interesting finding is that, at the same resource level, the correlation between overall WTA and functional trait stability showed an opposite trend to that of overall CTA and functional trait stability (Fig. 3a, b). Within the WTA, due to the predominance of positive WTA, the relationship between positive WTA and functional trait stability is the same as that of overall WTA, showing a positive relationship under low-resource conditions (R^2^ = 0.603 and 0.598, *P* < 0.001), but negative relationship under high resource conditions (R^2^ = 0.341 and 0.147, *P* < 0.001 and *P* < 0.05). However, for CTAs, it showed that functional trait stability decreased significantly with positive, negative, and overall CTA in low resources (R^2^ = 0.408–0.646, *P* < 0.001), but increased in high resources (R^2^ = 0.247–0.341, *P* < 0.01). These aforementioned linear regressions have also appeared between site-dependent network associations and soil functional stability (Figure S8).

### Drivers of soil functional trait stability

**Fig. 4:**
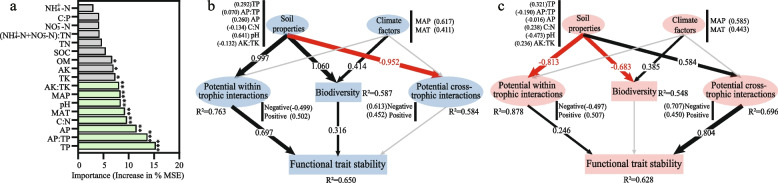
The direct and indirect effects of drivers on soil functional trait stability. **a** Mean predictor importance of factors on functional trait stability based on random forest analysis. Significant predictors revealed in the random forest analysis are marked with asterisks. *, *P* < 0.05; **, *P* < 0.01; ***, *P* < 0.001. Highly significant predictors (*P* < 0.01, columns marked with green) were selected for structural equation modeling. **b**, **c** Direct and indirect effects of driving factors on functional trait stabilities in low and high resource environments using Partial Least Squares Structural Equation Modeling (PLS-SEM), respectively. The ellipses represent the latent variables, and the rectangles represent the observed variables. The factor on the side of the latent variable is the observed variable, and the value in parentheses indicates the weight of the indicator. The black and red arrows in the PLS-SEM indicate positive and negative relationships, respectively, and gray arrows represent non-significant paths (*P* > 0.05). Numbers on the arrows are path coefficients, and the path widths represent the strength of path coefficient. OM, organic matter; AP, available phosphorus; AK, available potassium, NO_3_^−^-N, nitrate nitrogen, NH_4_^+^-N, ammonia nitrogen; (NO_3_^−^-N + NH_4_^+^-N): TN, the ratio of the sum of nitrate nitrogen and ammonia nitrogen to total nitrogen; AK:TK, the ratio of available potassium to total potassium; AP:TP, the ratio of available phosphorus to total phosphorus; C:N, the ratio of soil organic carbon to soil total nitrogen; C:P, the ratio of soil organic carbon to soil total phosphorus; MAT, mean annual temperature; MAP, mean annual precipitation

To determine the relative importance of resource-driven biodiversity and potential multi-trophic interactions on soil functional trait stability, we used Random Forest modeling first to separate and assess the important abiotic predictors of the functional trait stability (Fig. 4a). Then, the partial least structural equation modeling (PLS-SEMs) was used to quantify the contribution of important predictors to resource-driven biodiversity, potential multi-trophic interactions, and functional trait stability (Fig. 4b,c, Figure S9). The overall fitness of the resulting structural equation model is extremely strong (GoodFit = 0.719), suggesting that all of the important relationships were specified in the model (Table S6). Our PLS-SEMs explained 65% and 63% of the variance found in the functional trait stability of the low and high resource systems, respectively, after accounting for key ecosystem factors (*P* < 0.01) such as soil properties (TP, AP:TP, AP, C:N, pH, and AK:TK) and climate factors (MAP and MAT) (Fig. 4a). According to the scale of our investigation, we found that soil properties had a greater impact on belowground biodiversity and potential species interactions than the climate factors (Fig. 4b, c). When resources were low, soil properties influence functional trait stability indirectly via increases in biodiversity (path coefficient = 1.06, *P* = 0.002) and WTA (path coefficient = 0.997, *P* = 0.004). Although low resources depressed CTA, it had no direct effect on functional trait stability (*P* > 0.05). However, when soil resources were sufficient, soil properties shift to increase CTA but depress biodiversity and WTA. The variation in functional stability was explained entirely by potential multi-trophic interactions, especially CTA (path coefficient = 0.804, *P* = 0.006). Meanwhile, the direct effect of soil biodiversity on functional stability was not obvious. It is also consistent with the neutral relationship between biodiversity and functional trait stability across high-resource sites (Fig. 1e).

### Verification using experimental microcosm study

**Fig. 5:**
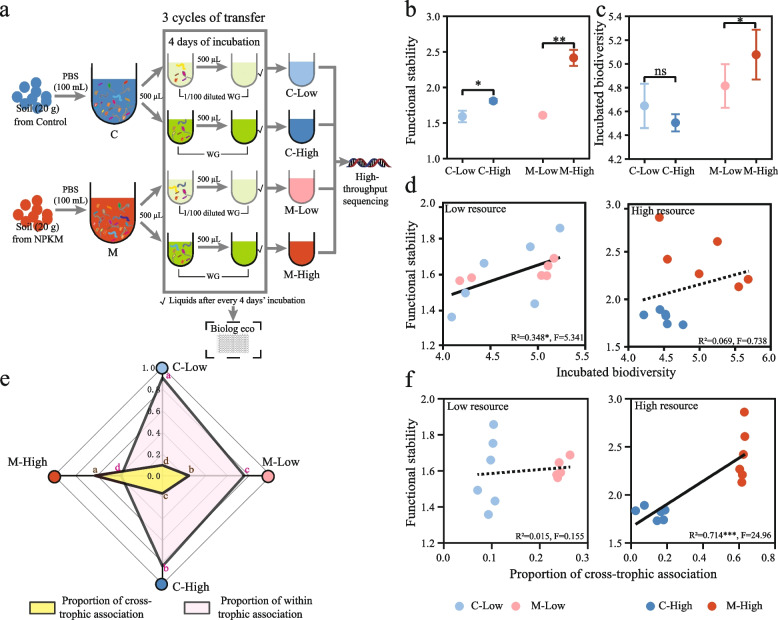
Experiment 1 of microcosm study. **a** Diagram of experiment 1 design. **b**, **c** The functional stability and incubated biodiversity after low and high resource incubations. Dots with error bars represent mean values and standard error of mean (*n* = 6) (Wilcoxon rank-sum test, *, *P* < 0.05; **, *P* < 0.01; ns, not significant). **d** Relationship between incubated biodiversity and functional stability after low and high resource incubations. The solid and dotted lines represent the significant and non-significant relationships, respectively. **e** Radar chart of the proportion of cross- and within trophic associations after high and low resource incubations. Different lowercase letters with brown and red colors represent significant differences of the proportion of cross-trophic and within trophic associations, respectively. **f** Relationship between cross-trophic association and functional stability after low and high resource incubations. WG, wheat grass medium; C-Low (light blue dots), soil suspension from C (control) was cultivated in low resource medium (1/100 diluted WG); C-High (dark blue dots), soil suspension from C (control) was cultivated in high resource medium (WG); M-Low (light red dots), soil suspension from M (NPKM) was cultivated in low resource medium; M-High (dark red dots), soil suspension from M (NPKM) was cultivated in high resource medium

**Fig. 6:**
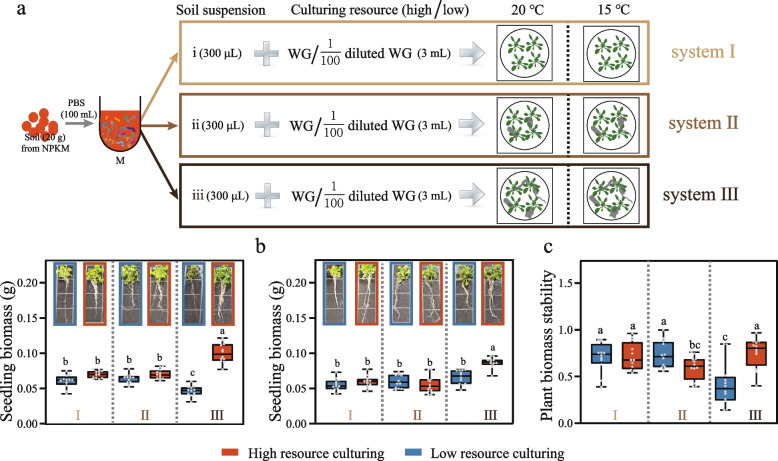
Experiment 2 of microcosm study. **a** Diagram of experiment 2 design. **b** Seedling biomass in different trophic-level culturing systems incubated at 20 °C (left) and 15 °C (right), respectively. System I, Control, axenic culture; system II: within trophic-level culture; system III: multi-trophic-level culture. Each sample includes four seedlings of *Arabidopsis thaliana*. **c** Plant biomass stability in different trophic-level cultures. The data are shown as the means ± standard deviations (*n* = 12). The error bars with different letters indicate significant differences as determined by one-way analysis of variance (ANOVA) followed by Tukey’s HSD test (*P* < 0.05)

Based on the field investigation, we found that resource supply resulted in a greater impact of cross-trophic associations on soil functional trait stability over large scales (Figs. 3 and 4). To confirm this pattern, we further conducted microcosm-scale, liquid culture experiments 1 and 2 with full-strength and diluted (1/100) WG (wheat grass) media. Soil organisms from the field soils of Yanting site were extracted in suspension, and then incubated in high (full-strength WG) and low nutrient incubation (1/100 diluted WG), respectively (Figs. 5a and 6a).

For experiment 1, we evaluated the effects of resource-driven multiple trophic associations and biodiversity on functional stability (Fig. 5a). Given continuous full-strength (high) resource incubation (microcosms of C-High and M-High), the functional stability increased 13.75% (*P* = 0.026) and 50.15% (*P* = 0.001) compared to those of 1/100 resource incubation (microcosms of C-Low and M-Low), respectively (Fig. 5b), suggesting abundant resources enhanced the stability of carbon metabolism. Whereas for the incubated biodiversity, only M-high increased 110% compared to M-Low (Fig. 5c, *P* = 0.047, Wilcoxon rank-sum test), and no difference was observed between C-Low and C-High (Fig. 5c, *P* > 0.05). Among these 4 microcosms, the proportion of CTA in high resource incubations (C-High and M-High) increased an average of 2 folds over those in low resource incubations (C-Low and M-Low), respectively (Fig. 5e, Figure S10, *P* < 0.001). As we expected, the incubated biodiversity showed a positive association with functional stability only when culturing resources were low (Fig. 5d, R^2^ = 0.35 and *P* < 0.05). Once the culturing resources become sufficient, no significant correlation was found indicating biodiversity cannot always support functional stability (Fig. 5d, R^2^ = 0.07 and *P* > 0.05). In contrast, the proportion of CTA transitioned from no relationship (R^2^ = 0.02 and *P* > 0.05) to a significant positive relationship (R^2^ = 0.71, and *P* < 0.001) with functional stability as the resource availability changed from low to high (Fig. 5f), suggesting the importance of CTA in stabilizing system multi-functionality in a resource-rich environment. Additionally, the functional stability in the microcosms was significantly linearly correlated with the functional trait stability measured from the field experiment (Figure S11, R^2^ = 0.84, F = 21.2 and *P* = 0.01), indicating consistent characteristics from the micro- and macro-scale studies.

To better understand whether the belowground functional stability enhancement finally cause the feedback to stabilize plant production, we established three different trophic-level micro-culture systems and planted *Arabidopsis thaliana* as the model in experiment 2 (Fig. 6a and Figure S12). Interestingly, system III with high resource availability always maintained maximum biomass, at both normal and low temperatures (Fig. 6b, *P* < 0.05). Whereas in systems I and II, plant biomass (Dataset S5) did not change with the different levels of resources at the same temperature (Fig. 6b, *P* > 0.05). Then, the stability of plant production was assessed by comparing the resistance of plant biomass to low temperature (Fig. 6c). In the absence of belowground organisms (system I), no difference in the stability of plant biomass was found between resource levels (*P* > 0.05). But once trophic-level organisms (bacteria) were co-cultured with plant roots in system II, low temperature reduced the stability of plant biomass 27.47% under the high resource condition (*P* < 0.05). In contrast, when multi-trophic-level organisms (including bacteria, fungi, protists and nematodes) were co-cultured in system III, a decrease in biomass stability was observed under the low resource condition. These results implied that multi-trophic associations in the presence of high nutrient concentration are the key drivers of the stability of micro-ecosystem functioning.

## Discussion

Despite the fundamental importance of stabilizing ecosystem functions for agricultural sustainability, a clear understanding of what determines functional stability is still lacking [73, 74]. Belowground biodiversity [1, 8, 10, 11] and interactions between species [75, 76] play key roles in stabilizing ecosystem multi-functionality, but how they comprehensively affect functional stability in intensive agroecosystems remains largely unknown. Here, we argue that the magnitude of impacts of biodiversity and potential multi-trophic interactions between organisms on functional trait stability depended on the level of resource availability. To be more specific, biodiversity, and potential within trophic interactions in low resource availability, and potential cross-trophic interactions in high resource availability are the drivers for the stability of belowground functional traits.

Community response to resource alteration usually begins with individual physiological and metabolic processes, followed by species reordering, and finally species mortality and immigration [77]. In this study, we found that long-term resource limitation, in agricultural systems reduced soil biodiversity, while resource input showed the opposite trend (Fig. 1c). It seems to be contrary to the natural pattern that resource limitation increases the opportunities for speciation in the case of plant communities [78, 79]. Different from natural ecosystems, single (P) or multiple nutrient (N, P, K) limitations caused by imbalanced fertilization in intensive agroecosystem directly leads to the extreme elemental stoichiometry in soil [80]. These important soil properties (e.g., P) will affect belowground biological functioning by the change of survival strategies of microbiomes and the loss of biodiversity [81, 82]. Conversely, high resource availability maintained soil biodiversity (Fig. 1c). It should be noted that the high resource mentioned in this study does not refer to nutrient enrichment caused by excessive fertilization. Actually, the amount of resource input, taking nitrogen (N, < 18 g N m^−2^ year^−1^) as the example in this study, is similar to “low N input” (≤ 16 g N m^−2^ year^−1^) standard in other studies, which showed “high N input (≥ 32 g N m^−2^ year^−1^)” negatively affect bacterial diversity [83]. Additionally, the types of resources, especially organic fertilizers, which have high heterogeneity in their nutrient composition, can provide opportunities for different species coexistence through niche differentiation facilitation [25].

Consistent with Pennekamp et al. [84], our field survey and microcosm study both supported that biodiversity can increase overall ecosystem stability when biodiversity is low in resource-limited conditions (Fig. 1d,e). A similar positive biodiversity-functional trait stability relationship can be found when all samples are taken into account, regardless of resource levels (Fig. 1e). It was in line with a multi-continent natural terrestrial ecosystem investigation that soil biodiversity enhances the ability of the ecosystem to maintain multi-functionality [85]. When resources were sufficient, both in the field experiment and microcosm study, although biodiversity and functional trait stability were synergistically improved, their linear relationship was not significant (Fig. 1e) and the relative importance of biodiversity to functional trait stability in PLS-SEM model was not obviously (Fig. 4c). Similar results were obtained in our two independent experiments, pointing to the robustness of our findings. Similarly, neutral effects of diversity on the temporal variability of aquatic algal communities also occur in the nutrient-rich microcosms [86]. In fact, theoretical models show that the effect of biodiversity on ecosystem stability can be positive, neutral, and negative [87, 88]. Some ecologists argued that the mysterious relationship between biodiversity and ecosystem stability is due to the fact that biodiversity is one of the divers of this relationship [87]. Here, we suggested that in addition to biodiversity, there are other drivers that underpin functional stability. Resource alteration may be of importance for the magnitude of biodiversity and other drivers affecting functional stability.

**Fig. 7:**
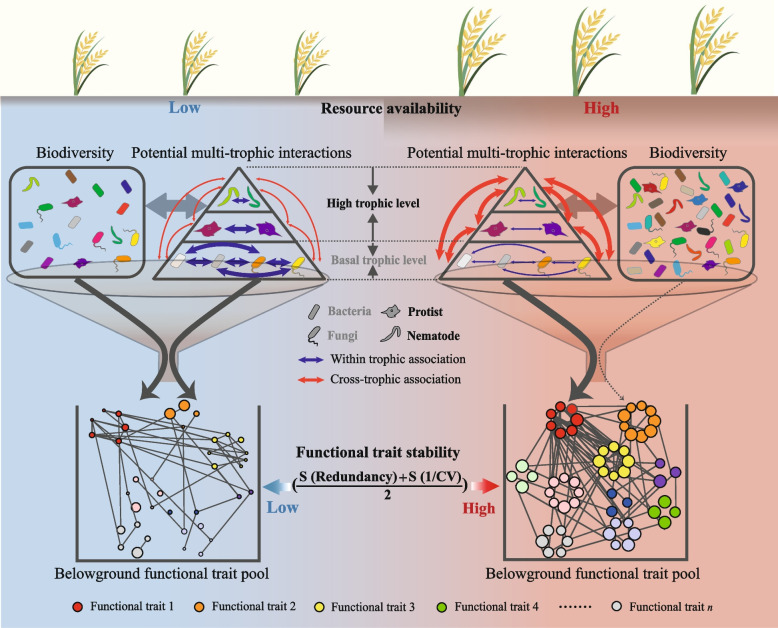
Schematic diagram of the potential regulation of belowground biodiversity and potential multi-trophic interactions to functional trait stability in low and high resource environments. Under the low resource condition, the biodiversity and species associations between the same trophic levels jointly affect the functional trait stability; while under the high resource condition, cross-trophic associations determine the functional trait stability. The boxes under the arrows indicate the functional trait pool. Circles with different colors indicate different functional traits. The size of the circle represents the coefficient of variation (CV) of the functional trait. The larger the circle, the greater the 1/CV. 1/CV is obtained by the ratio of the mean (*μ*) to the standard deviation (*σ*) of frequency of every functional trait. The larger the average value of standardized redundancy and 1/CV, the greater connectivity and stability of the functional traits

Chronic resource alteration also affected the interactions among species, the patterns and strengths of which strongly link to ecosystem stability [24, 75]. Using co-occurrence networks, a promising approach to investigate various types of potential interactions between organisms [8, 89], we found that when resources were limited, associations within the same trophic group, especially positive associations, enhanced the effect on the stability of multi-functionality (Fig. 3a, Fig. 4b, Figure S8a). This is easy to understand, as chronic resource depletion will push soil basal trophic-level decomposers, especially oligotrophic taxa, to cooperate closely to metabolize refractory organic substrates for survival [90]. The facilitation of different functional traits among small size species maintained the belowground multi-functionality. In parallel, the slow turnover of low levels of resources reduced energy fluxes to upper trophic organisms in the trophic pyramid [91] (Fig. 7), resulting in a decline in populations of mid-level consumers and weakening the ability of top-down forces to modulate functioning in the community [23] (Fig. 3b, Fig. 4b,c, and Figure S8d-f).

In contrast, nutrient supplementation led to pronounced competitive interactions between bacterial species [24]. This can be reflected by the more negative within trophic associations in high resource environments (Fig. 2b). As a result, microbial R-strategists would occupy the habitat niche through the rapid proliferation. However, the rapid growth of prey (R-strategists in particular) would subsequently increase the population of their predators (upper trophic level), due to the energy flow toward different trophic levels [22, 92]. Therefore, the increased proportion of cross-trophic associations were observed in both field and microcosm under high resource conditions (Fig. 2c and Fig. 5e). From the adjacent pairs of trophic-level perspective, theoretically, increased cross-trophic associations would provide a heterogeneous niche for more functional redundant species to coexist [93, 94] and should stabilize multi-functionality [13, 95] In line with this, the positive impact of cross-trophic associations on functional trait stability becomes prominent when resources are high according to the PLS-SEM modeling (Fig. 4c). More importantly, this positive belowground effect could be passed up to support the stability of plant biomass (Fig. 6b,c), suggesting the importance of overlooked soil–plant functional feedbacks in agro-ecosystems [96].

Different species in the community co-evolved to control ecosystem stability through multiple drivers such as microbial biodiversity and composition, microbiome complexity, and multi-trophic species interactions [8, 10, 75, 97]. Here, we found that the magnitude of each driver affecting belowground functional trait stability relies on soil resource status (Fig. 7). Frequent excessive resource inputs have been used to support global productivity at the expense of the decline of ecosystem stability and the loss of biodiversity [92, 97]. However, intensive planting with resource limitations, the other extreme, also destabilizes community function. Our results imply the importance of scheduled and quantitative resource complementation in maintain soil biodiversity and potential multi-trophic interactions for stabilizing functions of sustainable and healthy agro-ecosystems.

## Conclusions

Our results demonstrated that resource availability controlled the soil functional trait stability through mediating belowground biodiversity and potential multi-trophic interactions. Potential within trophic interactions and soil biodiversity together determined the functional trait stability when resource availability was low. High resource availability increased the potential cross-trophic interactions which greatly support the stability of functional traits. This pattern across agro-ecosystems has also appeared in two fine-scale microcosm systems. Our findings will benefit new policies and strategies of resource application for the sustainable intensification of agriculture.

## Supplementary Information

**Additional file 1: Figure S1. **Locations of soil sampling sites from Chinese National Ecosystem Research Network (CERN) in China. **Figure S2. **The pairwise correlation between the biodiversity and Bioref (Biorichness, Bioshannon, and Bioevenness). **Figure S3.** The relationship between biodiversity and richness, Shannon, and evenness index of soil bacterial, fungal, protist and nematode communities. **Figure S4. **The construction of site-dependent co-occurrence networks of soil organisms (including bacteria, fungi, protists, and nematodes) and proportions of multi-trophic association types in low and high available resource environments. **Figure S5. **Changes in average well color development (AWCD) (96 hours) with different resource level incubations in microcosm study. **Figure S6. **The pairwise correlation between diversities of single groups (including bacteria, fungi, protists, and nematodes) and functional trait stability. **Figure S7. **Proportions of within trophic (a) and cross-trophic (b) associations in low and high available resource environments based on site-dependent networks. **Figure S8.** Types of trophic association effects on soil functional trait stability across low and high resource available environments. **Figure S9. **A priori structural equation model including direct and indirect effects of resource availability, biodiversity, and within and cross-trophic associations on functional trait stability. **Figure S10. **The (bacterial, fungal, protist, and nematode) co-occurrence networks of biomes in the microcosm study.  **Figure S11. **Linear relationship between functional trait stability in field investigation and temporal functional stability in the experiment 1 of microcosm study. **FigureS12. **Picture showing 18-day-old *Arabidopsis *plants grown in different trophic level cultures (System I, Control, axenic culture; system II: within trophic level culture; system III: across trophic level culture) at 15 and 20 C. **Table S1. **Details of experimental field sites. **Table S2. **Soil chemical properties of low and high resource availability environments at each sampling site. **Table S3. **Indicator weight of each experimental site and calculation formula of indicator score. **Table S4. **Primer information for bacteria, fungi, protists, and nematodes. **Table S5. **Classification of potential species interaction types among belowground organisms (bacteria, fungi, protists, and nematodes). **Table S6. **Fitting index of Partial Least Squares Structural Equation Modeling (PLS-SEM). **Table S7. **Properties of ecological co-occurrence networks of soil cross-biome.

**Additional file 2.** 

**Additional file 3.**

**Additional file 4.**

**Additional file 5.**

**Additional file 6.**

## Data Availability

Data used in this work are available from the corresponding authors upon request. All data of bacterial, fungal, protist, and nematode sequencing, including samples that we exclude from the analysis, were deposited at The National Center for Biotechnology Information (NCBI) with the accession numbers PRJNA747872, PRJNA747891, PRJNA747906, and PRJNA836018, respectively. For the microcosm experiment, the accession numbers of bacterial, fungal, protist, and nematode sequences are PRJNA748514, PRJNA748515, PRJNA748517, and PRJNA748518, respectively. The R code is available from the GitHub Repository (https://github.com/Xingxingyueyang/Code). The unrarefied operational taxonomic unit table of bacteria, fungi, protists, and nematodes used in this study includes taxonomic information shown in Dataset S1. The unrarefied operational taxonomic unit table of bacteria, fungi, protists, and nematodes in microcosmic experiment 1 includes taxonomic information presented in Dataset S2. The frequency information of 10 potential functional genes in high and low resource environments of each site was deposited in Dataset S3. Biolog EcoPlate results after three incubations were deposited in Dataset S4. The biomass of 18-day-old *Arabidopsis* plants in different trophic-level cultures at 15 and 20 ℃ were shown in Dataset S5.

## References

[1] Delgado-baquerizo M, Reich PB, Trivedi C, Eldridge DJ, Abades S, Alfaro FD (2020). Ecosystem functions across biomes. Nat Ecol Evol.

[2] Tsiafouli MA, Thébault E, Sgardelis SP, de Ruiter PC, van der Putten WH, Birkhofer K (2015). Intensive agriculture reduces soil biodiversity across Europe. Glob Change Biol.

[3] Dai Z, Liu G, Chen H, Chen C, Wang J, Ai S (2020). Long-term nutrient inputs shift soil microbial functional profiles of phosphorus cycling in diverse agroecosystems. ISME J.

[4] Fan K, Delgado-Baquerizo M, Guo X, Wang D, Zhu Y guan, Chu H. Biodiversity of key-stone phylotypes determines crop production in a 4-decade fertilization experiment. ISME J. 2020;15:550–561 10.1038/s41396-020-00796-8 PMC8027226 33028975

[5] Wang X, Whalley WR, Miller AJ, White PJ, Zhang F, Shen J (2020). Sustainable cropping requires adaptation to a heterogeneous rhizosphere. Trends Plant Sci.

[6] Lidicker WZ (1979). A Clarification of interactions in ecological systems. Bioscience.

[7] Faust K, Raes J (2012). Microbial interactions: from networks to models. Nat Rev Microbiol.

[8] Wagg C, Schlaeppi K, Banerjee S, Kuramae EE, van der Heijden MGA (2019). Fungal-bacterial diversity and microbiome complexity predict ecosystem functioning. Nat Commun.

[9] Wagg C, Bender SF, Widmer F, Van Der Heijden MGA (2014). Soil biodiversity and soil community composition determine ecosystem multifunctionality. Proc Natl Acad Sci U S A.

[10] Delgado-Baquerizo M, Maestre FT, Reich PB, Jeffries TC, Gaitan JJ, Encinar D (2016). Microbial diversity drives multifunctionality in terrestrial ecosystems. Nat Commun.

[11] Soliveres S, Van Der Plas F, Manning P, Prati D, Gossner MM, Renner SC (2016). Biodiversity at multiple trophic levels is needed for ecosystem multifunctionality. Nature.

[12] Buzzard V, Michaletz ST, Deng Y, He Z, Ning D, Shen L (2019). Continental scale structuring of forest and soil diversity via functional traits. Nat Ecol Evol.

[13] Schuldt A, Assmann T, Brezzi M, Buscot F, Eichenberg D, Gutknecht J (2018). Biodiversity across trophic levels drives multifunctionality in highly diverse forests. Nat Commun.

[14] Laliberté E, Zemimik G, Turner BL (2014). Environmental filtering explains variation in plant diversity along resource gradients. Science.

[15] Fiore-Donno AM, Richter-Heitmann T, Bonkowski M (2020). Contrasting responses of protistan plant parasites and phagotrophs to ecosystems, land management and soil properties. Front Microbiol.

[16] Zhao ZB, He JZ, Geisen S, Han LL, Wang JT, Shen JP (2019). Protist communities are more sensitive to nitrogen fertilization than other microorganisms in diverse agricultural soils. Microbiome.

[17] Bongiorno G, Bodenhausen N, Bünemann EK, Brussaard L, Geisen S, Mäder P (2019). Reduced tillage, but not organic matter input, increased nematode diversity and food web stability in European long-term field experiments. Mol Ecol.

[18] Emery SM, Reid ML, Bell-Dereske L, Gross KL (2017). Soil mycorrhizal and nematode diversity vary in response to bioenergy crop identity and fertilization. GCB Bioenergy.

[19] Raes J, Bork P (2008). Molecular eco-systems biology: towards an understanding of community function. Nat Rev Microbiol.

[20] James GA, Beaudette L, Costerton JW (1995). Interspecies bacterial interactions in biofilms. J Ind Microbiol.

[21] Karakoç C, Clark AT, Chatzinotas A (2020). Diversity and coexistence are influenced by time-dependent species interactions in a predator–prey system. Ecol Lett.

[22] Geisen S, Hu S, dela Cruz TEE, Veen (Ciska) G. F. (2021). Protists as catalyzers of microbial litter breakdown and carbon cycling at different temperature regimes. ISME J.

[23] Wardle DA (2006). The influence of biotic interactions on soil biodiversity. Ecol Lett.

[24] Ratzke C, Barrere J, Gore J (2020). Strength of species interactions determines biodiversity and stability in microbial communities. Nat Ecol Evol.

[25] Hartmann M, Frey B, Mayer J, Mäder P, Widmer F (2015). Distinct soil microbial diversity under long-term organic and conventional farming. ISME J.

[26] Su JQ, Ding LJ, Xue K, Yao HY, Quensen J, Bai SJ (2015). Long-term balanced fertilization increases the soil microbial functional diversity in a phosphorus-limited paddy soil. Mol Ecol.

[27] Matz C, Jürgens K, Ecol M (2003). Interaction of nutrient limitation and protozoan grazing determines the phenotypic structure of a bacterial community.

[28] Rüger L, Feng K, Dumack K, Freudenthal J, Chen Y, Sun R (2021). Assembly patterns of the rhizosphere microbiome along the longitudinal root axis of maize (Zea mays L.). Front Microbiol.

[29] Schulz-Bohm K, Geisen S, Wubs ERJ, Song C, De Boer W, Garbeva P (2017). The prey’s scent - Volatile organic compound mediated interactions between soil bacteria and their protist predators. ISME J.

[30] Fonseca CR, Ganade G (2001). Species functional redundancy, random extinctions and the stability of ecosystems. J Ecol.

[31] Ferrer M, Martins dos Santos  VAP, Ott SJ, Moya A (2014). Gut microbiota disturbance during antibiotic therapy: a multi-omic approach. Gut Microbes.

[32] Louca S, Polz MF, Mazel F, Albright MBN, Huber JA, O’Connor MI (2018). Function and functional redundancy in microbial systems. Nat Ecol Evol.

[33] Wang Y, Cadotte MW, Chen Y, Fraser LH, Zhang Y, Huang F (2019). Global evidence of positive biodiversity effects on spatial ecosystem stability in natural grasslands. Nat Commun.

[34] Escalas A, Hale L, Voordeckers JW, Yang Y, Firestone MK, Alvarez-Cohen L (2019). Microbial functional diversity: from concepts to applications. Ecol Evol.

[35] Doran JW, Parkin BT. Defining and assessing soil quality. In: Doran JW, Coleman DC, Bezdicek DF, Stewart BA, editors. Defining soil quality for a sustainable environment, vol 35. Madison: Soil Science Society of America, Inc., Special Publication; 1994. p. 1–21.

[36] Qi YB, Darilek JL, Huang B, Zhao YC, Sun WX, Gu ZQ (2009). Evaluating soil quality indices in an agricultural region of Jiangsu province China. Geoderma.

[37] CLQG. Cultivated Land Quality Grade (GB/T33469–2016). National Standards of People’s Republic of China. 2016.

[38] Thiergart T, Garrido-oter R, Agler M, Kemen E, Schulze-lefert P, Thiergart T (2018). Microbial interkingdom interactions in roots promote Arabidopsis survival article microbial interkingdom interactions in roots promote Arabidopsis survival. Cell.

[39] Bulgarelli D, Rott M, Schlaeppi K, Ver Loren van Themaat E, Ahmadinejad N, Assenza F (2012). Revealing structure and assembly cues for Arabidopsis root-inhabiting bacterial microbiota. Nature.

[40] Delgado-Baquerizo M, Bardgett RD, Vitousek PM, Maestre FT, Williams MA, Eldridge DJ (2019). Changes in belowground biodiversity during ecosystem development. Proc Natl Acad Sci U S A.

[41] Tilman D (1999). The ecological consequences of changes in biodiversity: a search for general principles. Ecology.

[42] Langfelder P, Horvath S (2012). Fast R functions for robust correlations and hierarchical clustering. J Stat Softw.

[43] Barberán A, Bates ST, Casamayor EO, Fierer N (2012). Using network analysis to explore co-occurrence patterns in soil microbial communities. ISME J.

[44] Lima-Mendez G, Faust K, Henry N, Decelle J, Colin S, Carcillo F (2015). Determinants of community structure in the global plankton interactome. Science.

[45] Ma B, Wang Y, Ye S, Liu S, Stirling E, Gilbert JA (2020). Earth microbial co-occurrence network reveals interconnection pattern across microbiomes. Microbiome.

[46] Jiao S, Yang Y, Xu Y, Zhang J, Lu Y (2020). Balance between community assembly processes mediates species coexistence in agricultural soil microbiomes across eastern China. ISME J.

[47] Ma B, Wang H, Dsouza M, Lou J, He Y, Dai Z (2016). Geographic patterns of co-occurrence network topological features for soil microbiota at continental scale in eastern China. ISME J.

[48] Benjamini Y, Krieger AM, Yekutieli D (2006). Adaptive linear step-up procedures that control the false discovery rate. Biometrika.

[49] Xiao X, Liang Y, Zhou S, Zhuang S, Sun B (2018). Fungal community reveals less dispersal limitation and potentially more connected network than that of bacteria in bamboo forest soils. Mol Ecol.

[50] Feng K, Zhang Y, He Z, Ning D, Deng Y (2019). Interdomain ecological networks between plants and microbes. Mol Ecol Resour.

[51] Bonkowski M (2019). Microcosm approaches to investigate multitrophic interactions between microbial communities in the rhizosphere of plants. Methods Rhizosphere Biol Res.

[52] Brussaard L, de Ruiter PC, Brown GG (2007). Soil biodiversity for agricultural sustainability. Agric Ecosyst Environ.

[53] Zak J, Willig M, Moorhead D, Wildman H (1994). Functional diversity of microbial communities: a quantitative approach. Soil Biol Biochem.

[54] Smalla K, Wachtendorf U, Heuer H, Liu W, Forney L (1998). Analysis of BIOLOG GN substrate utilization patterns by microbial communities. Appl Environ Microbiol.

[55] Tilman D, Reich PB, Knops JMH (2006). Biodiversity and ecosystem stability in a decade-long grassland experiment. Nature.

[56] Craven D, Eisenhauer N, Pearse WD, Hautier Y, Isbell F, Roscher C (2018). Multiple facets of biodiversity drive the diversity–stability relationship. Nat Ecol Evol.

[57] Chen Y, Sun R, Sun T, Chen P, Yu Z, Ding L (2020). Evidence for involvement of keystone fungal taxa in organic phosphorus mineralization in subtropical soil and the impact of labile carbon. Soil Biol Biochem.

[58] Hou S, Thiergart T, Vannier N, Mesny F, Ziegler J, Pickel B (2021). A microbiota–root–shoot circuit favours Arabidopsis growth over defence under suboptimal light. Nat Plants.

[59] De Storme N, Geelen D (2020). High temperatures alter cross-over distribution and induce male meiotic restitution in Arabidopsis thaliana. Commun Biol.

[60] Nottingham AT, Bååth E, Reischke S, Salinas N, Meir P (2019). Adaptation of soil microbial growth to temperature: using a tropical elevation gradient to predict future changes. Glob Change Biol.

[61] Gansfort B, Uthoff J, Traunspurger W (2018). Interactions among competing nematode species affect population growth rates. Oecologia.

[62] Bradford MA, Davies CA, Frey SD, Maddox TR, Melillo JM, Mohan JE (2008). Thermal adaptation of soil microbial respiration to elevated temperature. Ecol Lett.

[63] Orwin KH, Wardle DA (2004). New indices for quantifying the resistance and resilience of soil biota to exogenous disturbances. Soil Biol Biochem.

[64] Liaw A, Wiener M (2002). Classification and regression by randomForest. R News.

[65] Fortmann-Roe S. A3: Accurate, adaptable, and accessible error metrics for predictive. R Package Version 09. 2013.

[66] Henseler J, Ringle CM, Sinkovics RR (2009). The use of partial least squares path modeling in international marketing. Adv Int Mark.

[67] Wold H. editor. Soft modelling: the basic design and some extensions. Syst Indirect Obs. North-Holland, Amsterdam. 1982. Part II.

[68] Henseler J, Ringle CM, Sarstedt M. Using partial least squares path modeling in international advertising research: basic concepts and recent issues. Edward Elgar Publishing. 2012. p. 252–276.

[69] Dijkstra TK, Esposito  Vinzi V, Chin WW, Henseler J, Wang H (2010). Latent variables and indices: Herman Wold’s basic design and partial least squares. Handbook of partial least squares: concepts, methods and applications.

[70] Ringle CM, Wende S, Becker J-M. SmartPLS 3. SmartPLS GmbH: Boenningstedt. http://www.smartpls.com.

[71] Jörg H (2013). Marko, Sarstedt. Goodness-of-fit indices for partial least squares path modeling.

[72] Wetzels M, Odekerken-Schröder G, Oppen CV (2009). Using PLS path modeling for assessing hierarchical construct models: guidelines and empirical illustration.

[73] Hautier Y, Tilman D, Isbe F, Seabloom EW, Borer ET, Reich PB (2015). Anthropogenic environmental changes affect ecosystem stability via biodiversity. Science.

[74] Mougi A, Kondoh M (2012). Diversity of interaction types and ecological community stability. Science.

[75] De Ruiter PC, Neutel AM, Moore JC (1995). Energetics, patterns of interaction strengths, and stability in real ecosystems. Science.

[76] Thakur MP, Geisen S (2019). Trophic regulations of the soil microbiome. Trends Microbiol.

[77] Smith MD, Knapp AK, Collins SL (2009). A framework for assessing ecosystem dynamics in response to chronic resource alterations induced by global change. Ecology.

[78] Lambers H, Raven JA, Shaver GR, Smith SE (2008). Plant nutrient-acquisition strategies change with soil age. Trends Ecol Evol.

[79] Lambers H, Brundrett MC, Raven JA, Hopper SD. Plant mineral nutrition in ancient landscapes: high plant species diversity on infertile soils is linked to functional diversity for nutritional strategies. 2011;348:7–27.

[80] Saifullah Khan K, Georg JR (2019). Stoichiometry of the soil microbial biomass in response to amendments with varying C/N/P/S ratios. Biol Fertil Soils.

[81] Camargo AP, de Souza RSC, Jose J, Gerhardt IR, Dante RA, Mukherjee S (2023). Plant microbiomes harbor potential to promote nutrient turnover in impoverished substrates of a Brazilian biodiversity hotspot. ISME J.

[82] Wu X, Rensing C, Han D,  Xiao K-Q,, Dai Y, Tang Z (2022). Genome-resolved metagenomics reveals distinct phosphorus acquisition strategies between soil microbiomes. mSystems.

[83] Liu W, Jiang L, Yang S, Wang Z, Tian R, Peng Z (2020). Critical transition of soil bacterial diversity and composition triggered by nitrogen enrichment. Ecology.

[84] Pennekamp F, Pontarp M, Tabi A, Altermatt F, Alther R, Choffat Y (2018). Biodiversity increases and decreases ecosystem stability. Nature.

[85] Delgado-Baquerizo M, Reich PB, Trivedi C, Eldridge DJ, Abades S, Alfaro FD (2020). Multiple elements of soil biodiversity drive ecosystem functions across biomes. Nat Ecol Evol.

[86] Zhang Q, Zhang D (2006). Resource availability and biodiversity effects on the productivity, temporal variability and resistance of experimental algal communities. Oikos.

[87] Mccann KS (2000). The diversity–stability debate. Nature.

[88] Ives AR, Carpenter SR (2007). Stability and diversity of ecosystems. Science.

[89] Xiong W, Song Y, Yang K, Gu Y, Wei Z, Kowalchuk GA (2020). Rhizosphere protists are key determinants of plant health. Microbiome.

[90] Fierer N, Lauber CL, Ramirez KS, Zaneveld J, Bradford MA, Knight R (2012). Comparative metagenomic, phylogenetic and physiological analyses of soil microbial communities across nitrogen gradients. ISME J.

[91] Heath MR, Speirs DC, Steele JH (2014). Understanding patterns and processes in models of trophic cascades. Ecol Lett.

[92] Tilman D, Cassman KG, Matson PA, Naylor R, Stephen P (2002). Agricultural sustainability and intensive production practices. Nature.

[93] Cooke RSC, Bates AE, Eigenbrod F (2019). Global trade-offs of functional redundancy and functional dispersion for birds and mammals. Glob Ecol Biogeogr.

[94] Storch D, Bohdalková E, Okie J (2018). The more-individuals hypothesis revisited: the role of community abundance in species richness regulation and the productivity-diversity relationship. Ecol Lett.

[95] Isbell FI, Polley HW, Wilsey BJ (2009). Biodiversity, productivity and the temporal stability of productivity: patterns and processes. Ecol Lett.

[96] Kardol P, Martijn Bezemer T, Van Der Putten WH (2006). Temporal variation in plant-soil feedback controls succession. Ecol Lett.

[97] Tilman D, Balzer C, Hill J, Befort BL (2011). Global food demand and the sustainable intensification of agriculture. Proc Natl Acad Sci U S A.

