# Supplementary material for: Resource-dependent biodiversity and potential multi-trophic interactions determine belowground functional trait stability
Source: Microbiome. 2023 May 1;11:95. doi: 10.1186/s40168-023-01539-5 (PMC10150482; doi:10.1186/s40168-023-01539-5)
Supplement: Supplementary file 2 — Additional file 1: Figure S1. Locations of soil sampling sites from Chinese National Ecosystem Research Network (CERN) in China. Figure S2. The pairwise correlation between the biodiversity and Bioref (Biorichness, Bioshannon, and Bioevenness). Figure S3. The relationship between biodiversity and richness, Shannon, and evenness index of soil bacterial, fungal, protist and nematode communities. Figure S4. The construction of site-dependent co-occurrence networks of soil organisms (including bacteria, fungi, protists, and nematodes) and proportions of multi-trophic association types in low and high available resource environments. Figure S5. Changes in average well color development (AWCD) (96 hours) with different resource level incubations in microcosm study. Figure S6. The pairwise correlation between diversities of single groups (including bacteria, fungi, protists, and nematodes) and functional trait stability. Figure S7. Proportions of within trophic (a) and cross-trophic (b) associations in low and high available resource environments based on site-dependent networks. Figure S8. Types of trophic association effects on soil functional trait stability across low and high resource available environments. Figure S9. A priori structural equation model including direct and indirect effects of resource availability, biodiversity, and within and cross-trophic associations on functional trait stability. Figure S10. The (bacterial, fungal, protist, and nematode) co-occurrence networks of biomes in the microcosm study. Figure S11. Linear relationship between functional trait stability in field investigation and temporal functional stability in the experiment 1 of microcosm study. FigureS12. Picture showing 18-day-old Arabidopsis plants grown in different trophic level cultures (System I, Control, axenic culture; system II: within trophic level culture; system III: across trophic level culture) at 15 and 20 C. Table S1. Details of experimental field sites. Table S2. Soil c [file 40168_2023_1539_MOESM1_ESM.docx]

**Supplementary Information**

**Resource dependent biodiversity and** **potential** **multi-trophic interactions determine belowground functional trait stability**

Lingyue Zhu^1,2†^, Yan Chen^1†*^, Ruibo Sun^3^, Jiabao Zhang^1*^, Lauren Hale^4,5^, Kenneth Dumack^6^, Stefan Geisen^7,8^, Ye Deng^9,10^, Yinghua Duan^11^, Bo Zhu^12^, Yan Li^13^, Wenzhao Liu^14^, Xiaoyue Wang^1^, Bryan S. Griffiths^15^, Michael Bonkowski^6^, Jizhong Zhou^4^, Bo Sun^1*^

^1^ State Key Laboratory of Soil and Sustainable Agriculture, Institute of Soil Science, Nanjing 210008, China.

^2^ University of Chinese Academy of Sciences, Beijing 100049, China.

^3^ Anhui Province Key Laboratory of Farmland Ecological Conservation and Pollution Prevention, College of Resources and Environment, Anhui Agricultural University, Hefei 230036, China.

^4^ Institute for Environmental Genomics, University of Oklahoma, Norman, Oklahoma 73019, USA.

^5^ Agricultural Research Service (ARS), United States Department of Agriculture, Washington DC 20250, USA.

^6^ Terrestrial Ecology, Institute of Zoology, University of Cologne, Cologne 50674, Germany.

^7^ Laboratory of Nematology, Wageningen University & Research, Wageningen 6708 PB, The Netherlands.

^8^ Department of Terrestrial Ecology, Netherlands Institute of Ecology (NIOO-KNAW), Wageningen 6700AB, The Netherlands.

^9^ CAS Key Laboratory for Environmental Biotechnology, Research Center for Eco-Environmental Sciences, Chinese Academy of Sciences, Beijing, China

^10^ College of Resources and Environment, University of Chinese Academy of Sciences, Beijing 100081, China

^11^ Institute of Agricultural Resources and Regional Planning, Chinese Academy of Agricultural Sciences, Beijing 100081, China.

^12^ Institute of Mountain Hozards and Environment, Chinese Academy of Sciences, Chengdu 610041, China.

^13^ Xinjiang Institute of Ecology and Geography, Chinese Academy of Sciences, Urumqi 830011, China.

^14^ Institute of Soil and Water Conservation, Chine Academy of Sciences and Ministry of Water Resources, Yangling 712100, China.

^15^ SRUC, Crop and Soil System Research Group, West Mains Road, Edinburgh EH93JG, UK

^†^ These authors contributed equally: Lingyue Zhu, Yan Chen

^*^Corresponding authors: Yan Chen chenyan@issas.ac.cn; Bo Sun [bsun@issas.ac.cn](mailto:bsun@issas.ac.cn); Jiabao Zhang [jbzhang@issas.ac.cn](mailto:jbzhang@issas.ac.cn)

**Contents**

**Supplementary Methods**

**Supplementary Figure S1 to S12**

**Figure S1.** Locations of soil sampling sites from Chinese National Ecosystem Research Network (CERN) in China.

**Figure S2.** The pairwise correlation between the biodiversity and Bioref (Biorichness, Bioshannon, and Bioevenness).

**Figure S3.** The relationship between biodiversity and richness, Shannon, and evenness index of soil bacterial, fungal, protist and nematode communities.

**Figure S4.** The construction of site-dependent co-occurrence networks of soil organisms (including bacteria, fungi, protists, and nematodes) and proportions of multi-trophic association types in low and high available resource environments.

**Figure S5.** Changes in average well color development (AWCD) (96 hours) with different resource level incubations in microcosm study.

**Figure S6.** The pairwise correlation between diversities of single groups (including bacteria, fungi, protists, and nematodes) and functional trait stability.

**Figure S7.** Proportions of within trophic (a) and cross-trophic (b) associations in low and high available resource environments based on site-dependent networks.

**Figure S8.** Types of trophic association effects on soil functional trait stability across low and high resource available environments.

**Figure S9.** A priori structural equation model including direct and indirect effects of resource availability, biodiversity, and within and cross-trophic associations on functional trait stability.

**Figure S10.** The (bacterial, fungal, protist, and nematode) co-occurrence networks of biomes in the microcosm study.

**Figure S11.** Linear relationship between functional trait stability in field investigation and temporal functional stability in the experiment 1 of microcosm study.

**Figure S12.** Picture showing 18-day-old *Arabidopsis* plants grown in different trophic level cultures (System Ⅰ, Control, axenic culture; system Ⅱ: within trophic level culture; system III: across trophic level culture) at 15 and 20 ℃.

**Supplementary Table S1 to S7**

**Table S1.** Details of experimental field sites.

**Table S2.** Soil chemical properties of low and high resource availability environments at each sampling site.

**Table S3.** Indicator weight of each experimental site and calculation formula of indicator score.

**Table S4.** Primer information for bacteria, fungi, protists, and nematodes.

**Table S5.** Classification of potential species interaction types among belowground organisms (bacteria, fungi, protists, and nematodes).

**Table S6.** Fitting index of Partial Least Squares Structural Equation Modeling (PLS-SEM)

**Table S7.** Properties of ecological co-occurrence networks of soil cross-biome.

**Supplementary Methods**

**Soil properties were determined using standardized protocols.** Soil pH was detected by a pH meter using a soil/water ratio of 1:2.5 [1]. Soil organic matter was determined using the dichromate oxidization method [2]. Total nitrogen (TN) was determined by Kjeldahl method [3]. Total phosphorus (TP) and total potassium (TK) was digested with HF-HClO4 and determined using the molybdenum-blue method[4] and atomic absorption spectrophotometer, respectively [5]. Soil available phosphorus (AP) was extracted with sodium bicarbonate and determined using the molybdenum-blue method [6]. Available potassium (AK) was extracted with ammonium acetate and detected by ﬂame photometry [6]. Ammonium nitrogen (NH_4_^+^) was determined through 0.01 mol L^-1^ calcium chloride extraction–ultraviolet spectrophotometry, and nitrate nitrogen (NO_3_^-^) was determined by saturated calcium sulfate extraction–spectrophotometry [6]. C:N was obtained by calculating the ratio of soil organic carbon to total nitrogen, and C:P was obtained by the ratio of soil organic carbon to total phosphorus.

**Soil DNA extraction and quality inspection**

To meet the requirements of the high-throughput sequencing and functional gene-array-based high-throughput (Geochip) analysis, a total of 1000 ng of DNA was extracted from each soil sample. Soil DNA was extracted using the Powersoil DNA Isolation Kit (MoBio Laboratories, USA) according to the manufacturer's instructions. Extracted DNA was quantified and qualified using a Nanodrop ND-1000 spectrophotometer at 260/280 and 260/230 ratios ≥1.8 (NanoDrop Technologies, Delaware, USA). 200 nanogram of DNA was used for high-throughput sequencing of bacterial, fungal, protist, and nematode community, while another 800 ng of DNA was used for functional gene analysis.

**High-throughput sequencing**

Taxonomic profiles of soil bacterial, fungal, protist, and nematode communities were determined using amplicon sequencing on the Illumina MiSeq platform. A portion of the bacterial 16S rRNA, fungal ITS, and protist and nematode 18S rRNA genes were sequenced using the respective specific primer sets with 7-bp barcode sequence to identify samples (Supplementary Table S4). Bioinformatics processing was performed according to previous studies [7–10]. Briefly, the paired-end raw reads were trimmed using Trimmonatic [11] and merged using fastq-join with the default parameters [12]. Then the primer sequences were removed using cutadapt [13], and chimeric reads and singletons were filtered out using VSEARCH [14]. High-quality sequences were clustered into operational taxonomic units (OTUs) with a similarity threshold of 97% using VSEARCH [14]. Taxonomic assignments of the OTUs were performed using RDP Classifier [15] (Dataset S1 and S2). After removing OTUs not assigned as bacteria, fungi, protists, or nematodes, all samples were rarefied to 25000 (bacteria, 16S rRNA gene), 12000 (fungi, ITS), 1100 (protists, 18S rRNA gene), and 1300 (nematodes, 18S rRNA gene) sequences to ensure an even sampling depth within each belowground group of organisms.

**GeoChip hybridization**

To assess potential soil functions we used GeoChip 5.0M [16,17], a functional gene-array-based high-throughput technology designed for profiling the functional structure, diversity, metabolic potential and dynamics of microbial communities [18]. GeoChip 5.0M enables detection and quantification of the abundance of >36,500 functional genes from 1,447 gene families involved in the biogeochemical cycling of C, N, P, and S, and other functional categories [19]. Geochip has been widely used in various studies to understand the functional potential of complex communities across different environments [16,20,21]. In this study, genes involved in 10 functional groups, including C, N, P, and S cycling, electron transfer and stress response were classified, and their frequency calculated. Sequence retrieval, probe design, microarray construction and imaging were described previously [18]. Details for DNA labeling, hybridization, image processing and data processing were performed as described in Cheng et al. [22].


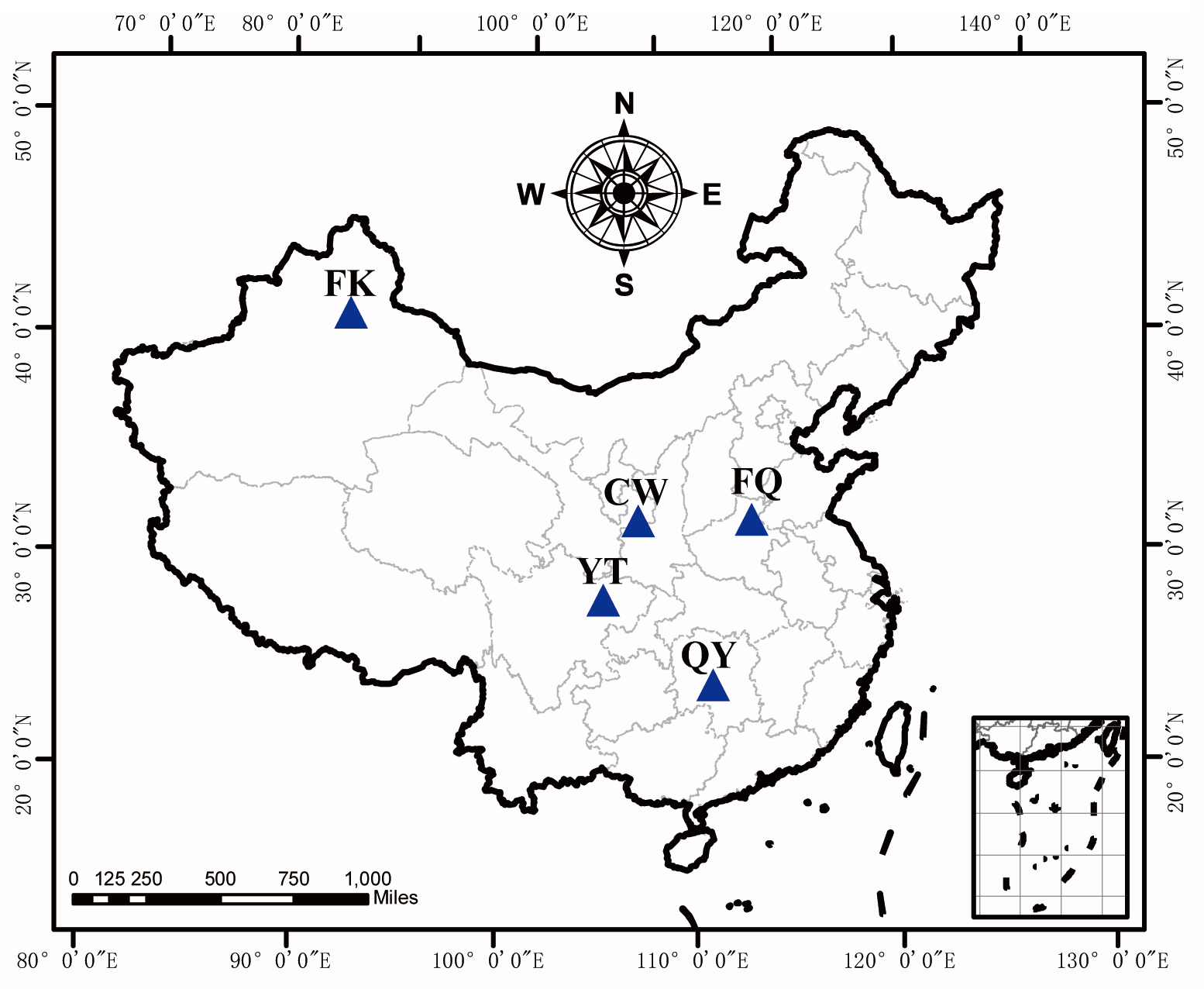


**Figure S1.** Locations of soil sampling sites from Chinese National Ecosystem Research Network (CERN) in China (Supplementary Table 1). FQ, Fengqiu; CW, Changwu; YT, Yangting; QY, Qiyang; FK, Fukang.
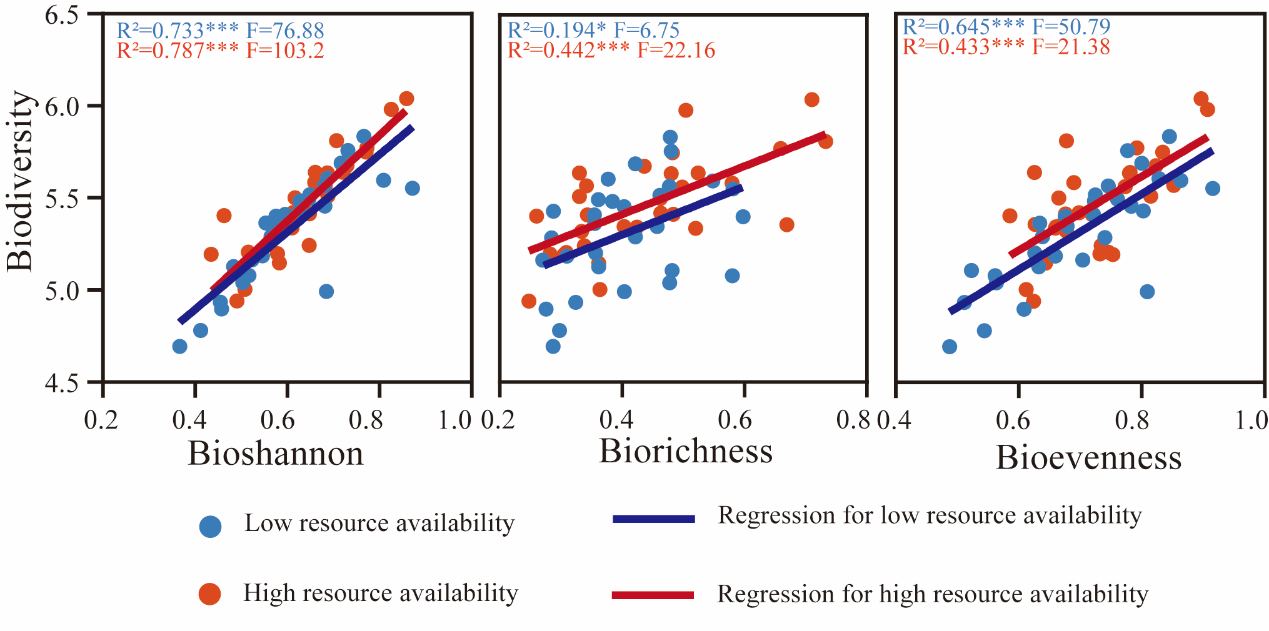


**Figure S2.** The pairwise correlation between the biodiversity and Bioref (Biorichness, Bioshannon, and Bioevenness). For the specific calculation method and meaning of each indicator, refer to the Method section of belowground biodiversity index. Blue dots represent samples from low resource treatments, while red dots represent samples from high resource treatments. Blue and red lines represent the significant relationships in low and high resource environments, respectively. *, *P* <0.05; **, *P* <0.01; ***, *P* <0.001.


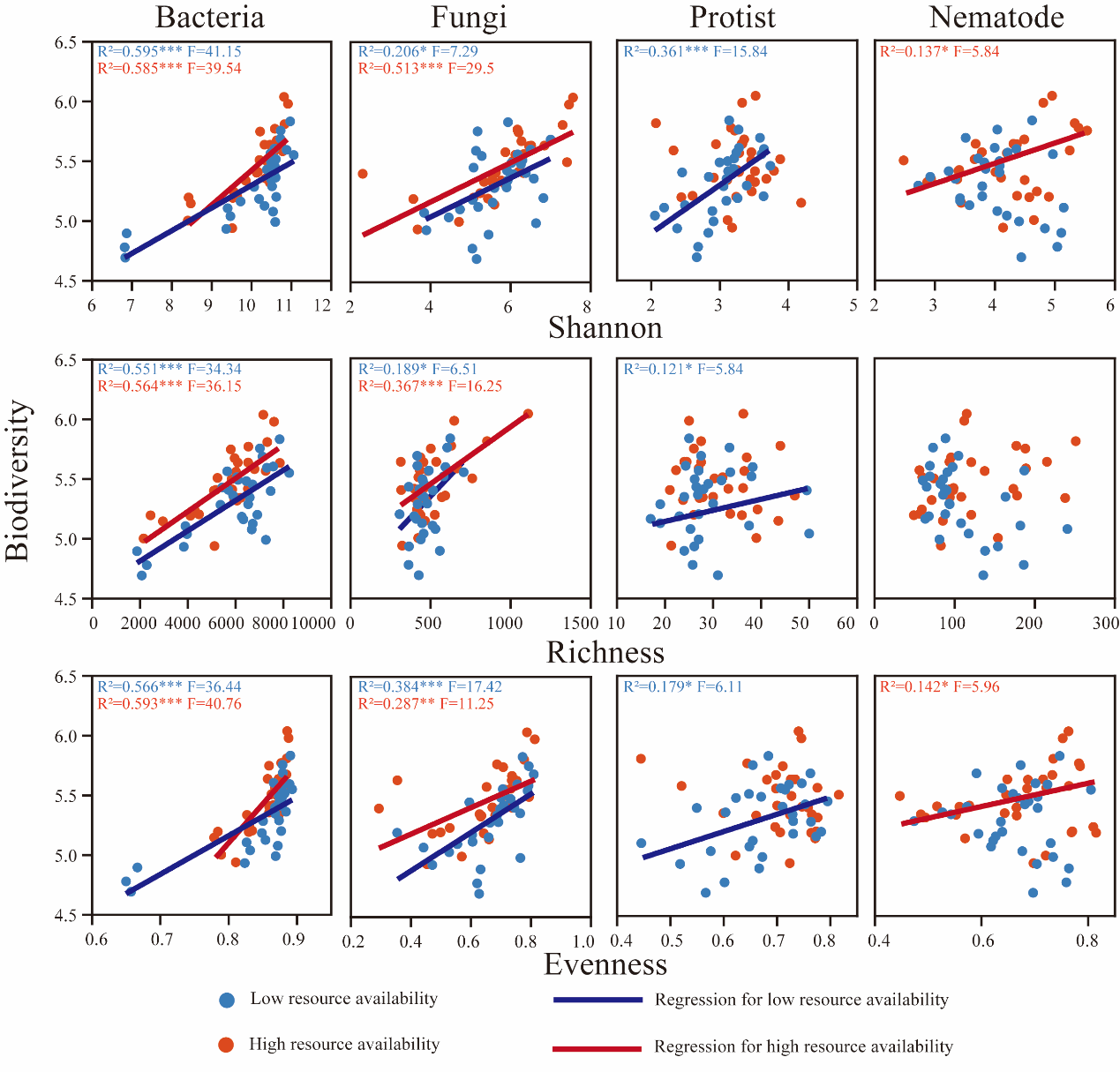


**Figure S3.** The relationship between biodiversity and richness, Shannon, and evenness index of soil bacterial, fungal, protist and nematode communities. Blue dots represent samples from low resource sites, while red dots represent samples from high resource sites. Blue and red lines represent the significant relationships in low and high resource environments, respectively. *, *P* <0.05; **, *P* <0.01; ***, *P* <0.001.


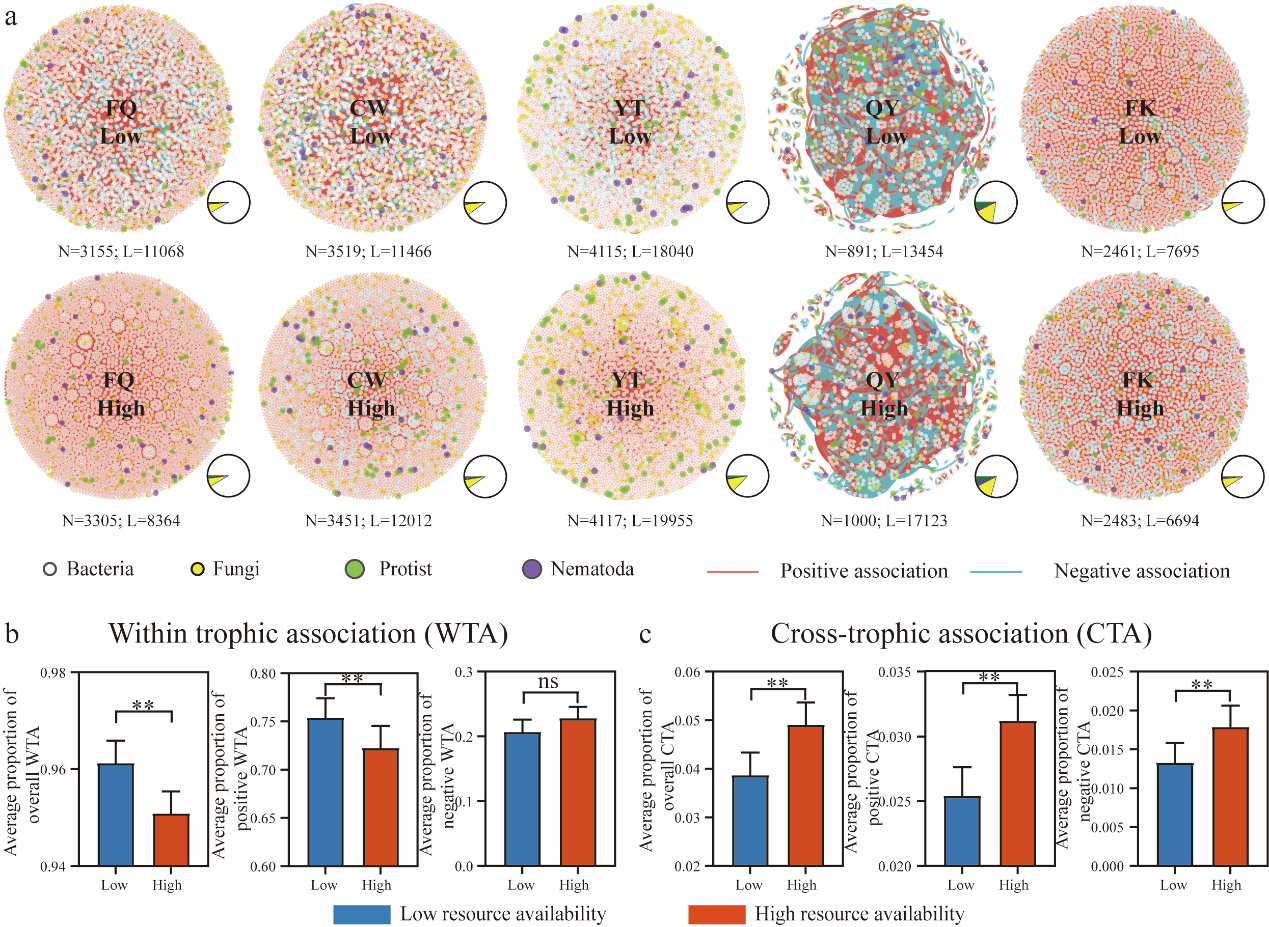


**Figure S4.** The construction of site-dependent co-occurrence networks of soil organisms (including bacteria, fungi, protists, and nematodes) and proportions of multi-trophic association types in low and high available resource environments. **a** Visualization of site-dependent co-occurrence networks across low and high resource availability treatments. A connection between two nodes represents a significant Spearman correlation (*P*<0.05). A red link represents positive association between two nodes, while a blue link represents negative association. “N” and “L” under each network represent the number of nodes and links, respectively. Pie chart in the bottom right of each network indicates the nodes proportion of bacteria, fungi, protists, and nematodes in the coexistent organism. **b-c** Average proportion of within trophic (WTA) and cross-trophic associations (CTA) in low and high available resource based on site-dependent networks. Asterisks denote significant differences between metrics for low and high resource availability soils (*n*=30) (Wilcoxon rank-sum test, *, *P* <0.05; **, *P* <0.01; ns, not significant).


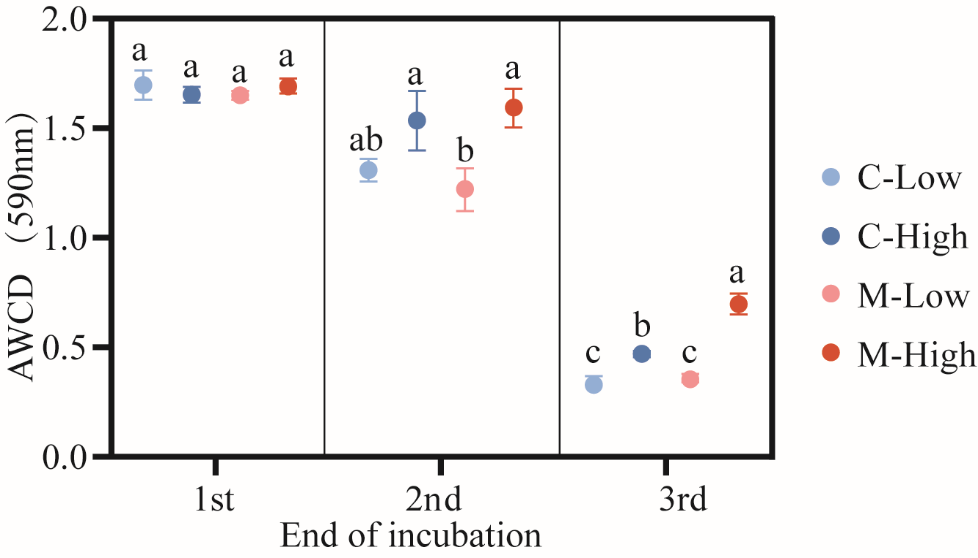


**Figure S5.** Changes in average well color development (AWCD) (96 hours) with different resource level incubations in microcosm study. Error bars with different letters represent significant differences of individual parameters among the three stands by *Tukey’s* HSD post hoc tests. (Please refer to Fig. 5 for abbreviations).


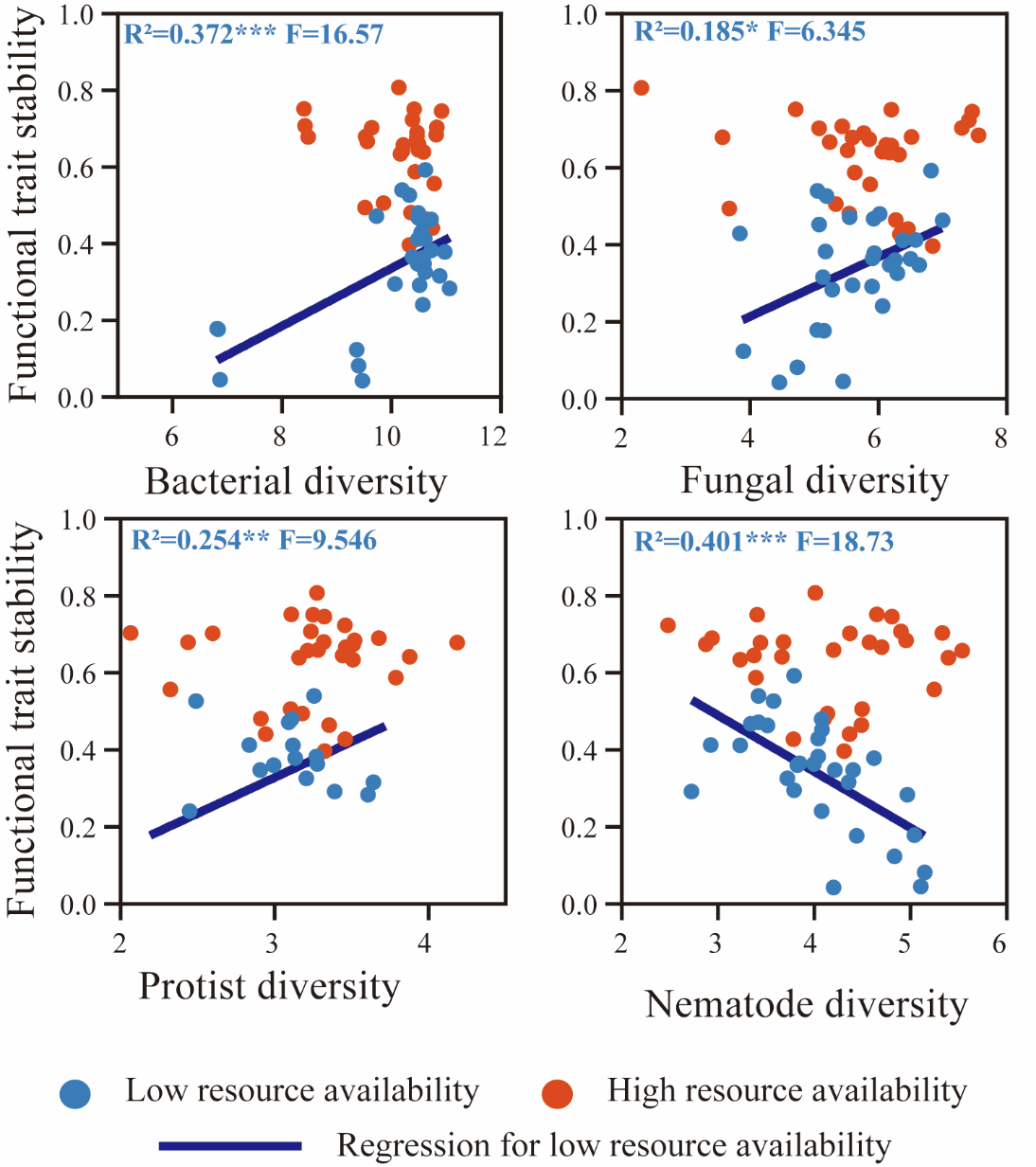


**Figure S6.** The pairwise correlation between diversities of single groups (including bacteria, fungi, protists, and nematodes) and functional trait stability. The solid blue line represents the significant relationships between diversity of single group and functional trait stability in samples with low resource availability. No significant correlations were found in high resource environments.


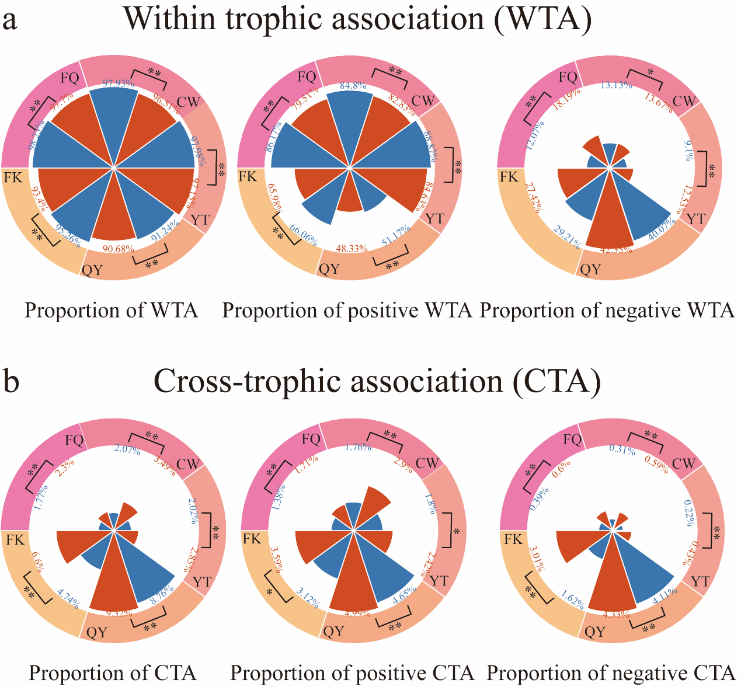


**Figure S7.** Proportions of within trophic (a) and cross-trophic (b) associations in low and high available resource environments based on site-dependent networks. Asterisks denote significant differences between metrics for low and high resource availability soils within each site (*n*=6) (Wilcoxon rank-sum test, *, *P* <0.05; **, *P* <0.01; ns, not significant).


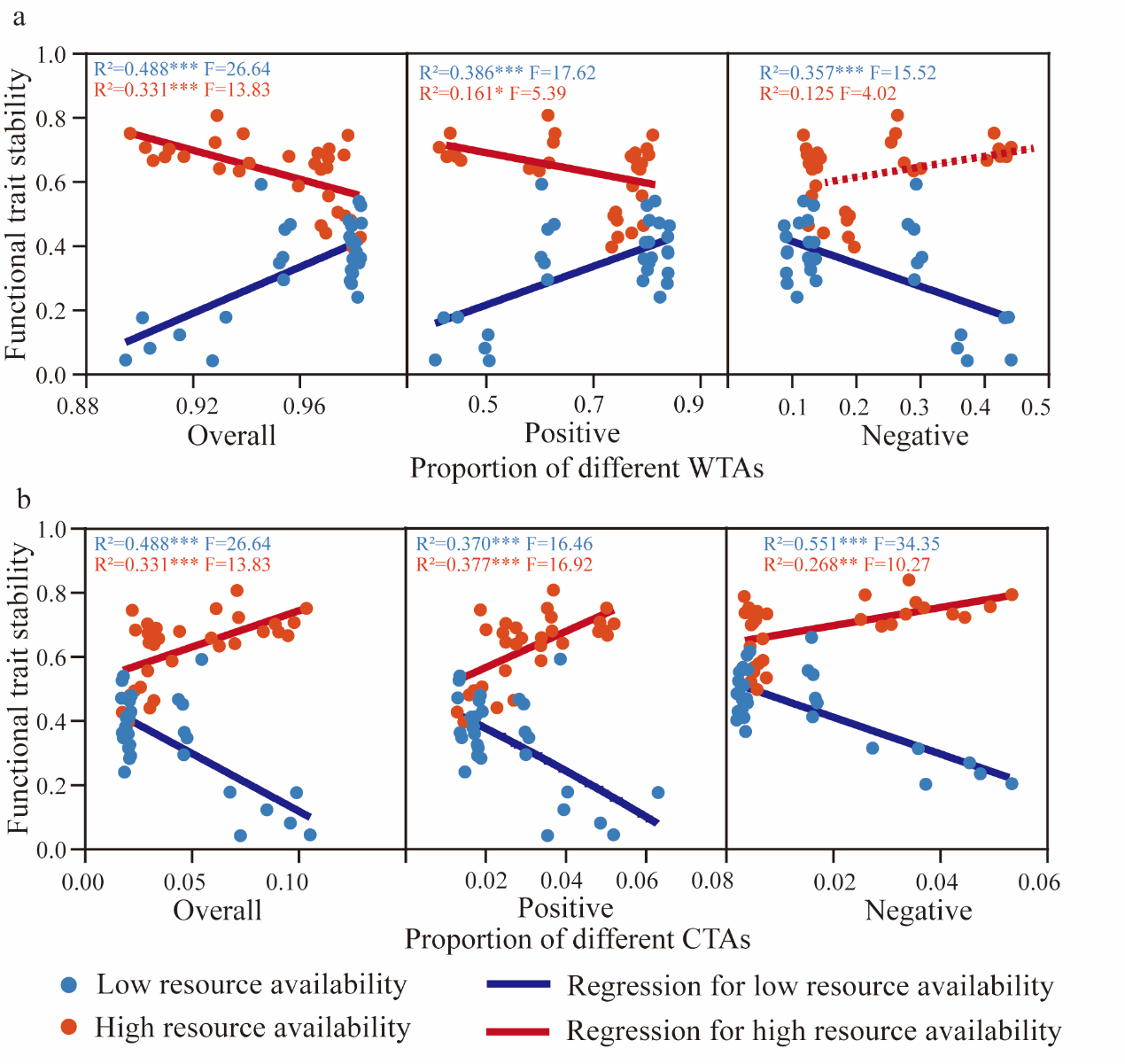
 **Figure S8.** Types of trophic association effects on soil functional trait stability across low and high resource available environments. Blue and red dots represent samples from low and high resource treatments, respectively. Solid and dashed lines represent the significant and non-significant linear relationships in low and high resource environments, respectively. WTA, within trophic association; CTA, cross-trophic association. *, *P* <0.05; **, *P* <0.01; ***, *P* <0.001.


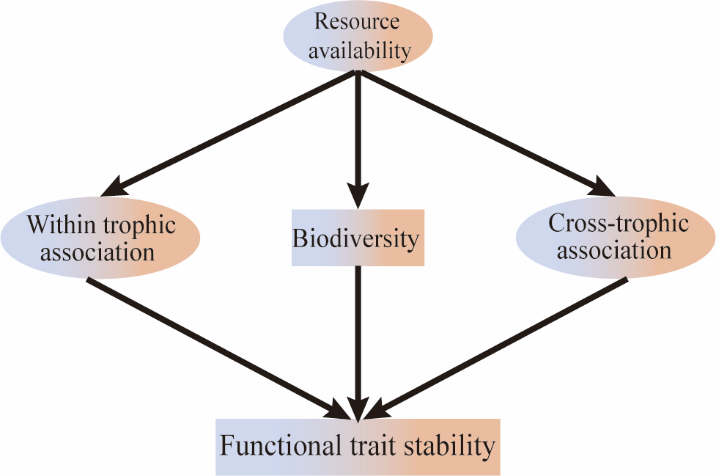


**Figure S9.** A priori structural equation model including direct and indirect effects of resource availability, biodiversity, and within and cross-trophic associations on functional trait stability.


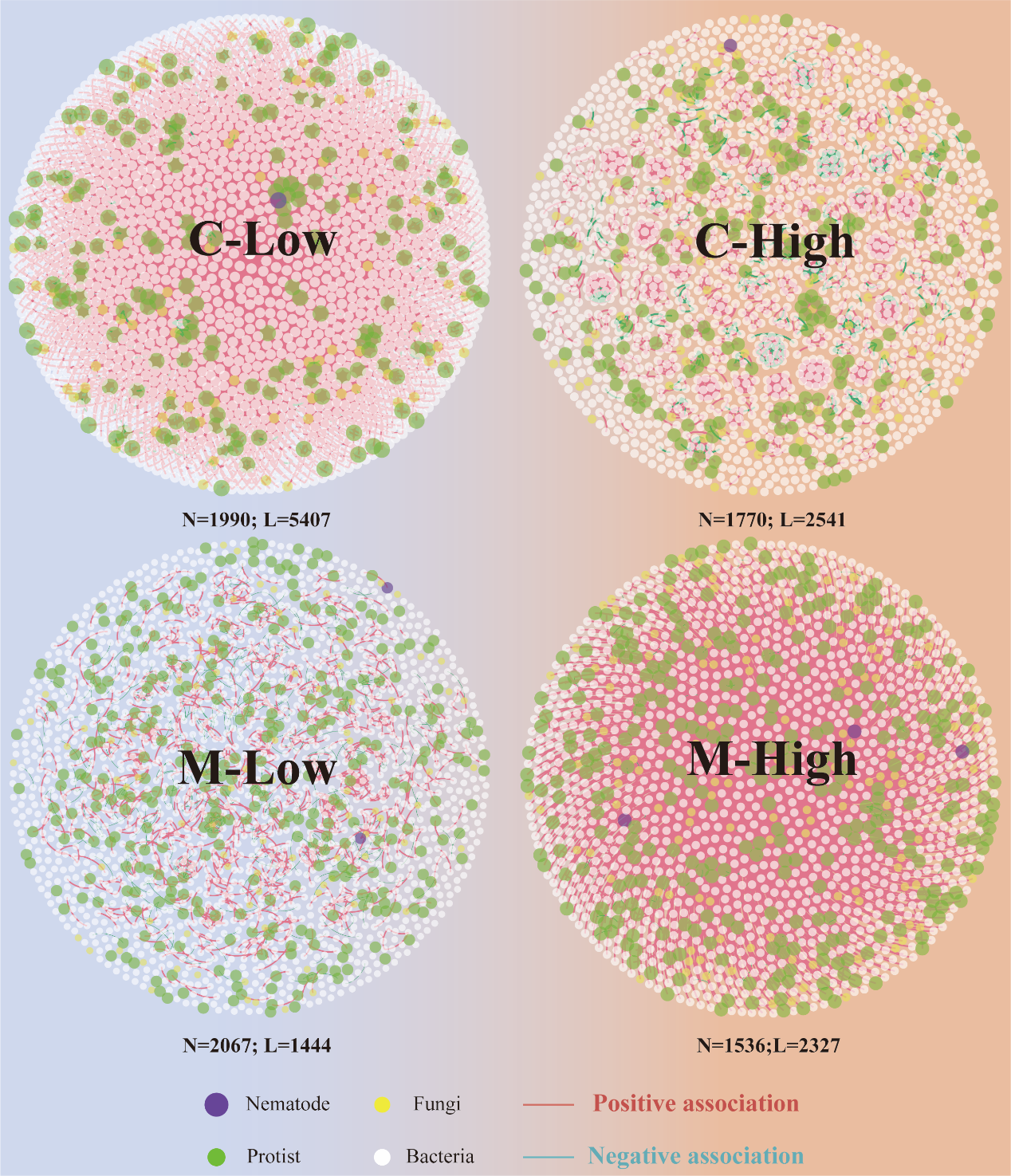
**Figure S10.** The (bacterial, fungal, protist, and nematode) co-occurrence networks of biomes in the microcosm study. A connection between two nodes (i.e., a link) represents the significant Spearman correlation. “N” and “L” under each network represent the number of nodes and links, respectively. A red link represents positive association between two nodes, while a blue link represents negative association. (Please refer to Fig. 5 for abbreviations).


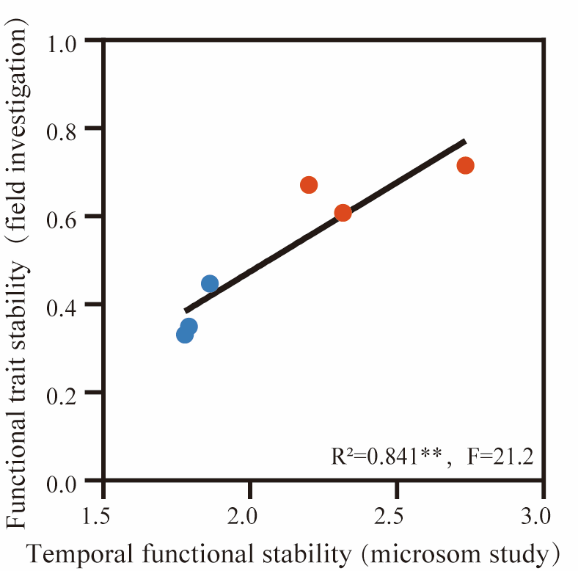


**Figure S11.** Linear relationship between functional trait stability in field investigation and temporal functional stability in the experiment 1 of microcosm study. **, *P* <0.01.


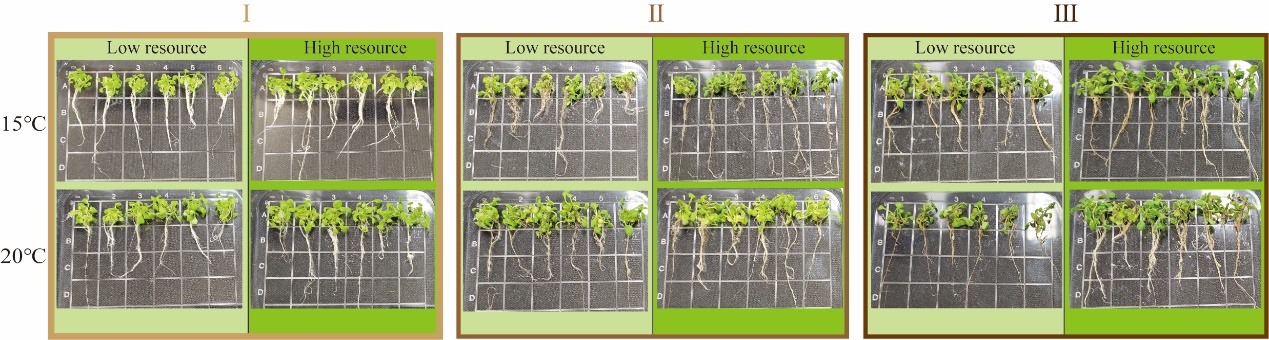


**Figure S12.** Picture showing 18-day-old *Arabidopsis* plants grown in different trophic level cultures (System Ⅰ, Control, axenic culture; system Ⅱ: within trophic level culture; system III: across trophic level culture) at 15 and 20 ℃. The low resource represents culture resource that was 1/100 diluted WG; high resource represents culture resource that was undiluted WG.

**Table S1.** Details of experimental field sites.

|  | **Province** | **Location** | **MT, MP** | **Soil taxonomy (USDA)** | **Planting** | **Treatments of low resource availability** | **Treatments of high resource availability** |
| --- | --- | --- | --- | --- | --- | --- | --- |
| FQ | Henan | 35°1′12″N, 114°31′48″E | 605mm, 15.24℃ | Inceptisol | Maize-  wheat  rotation | 1. Control:   No fertilizer input   1. NK:   N: urea (176.25 kg N ha^-1^)  K: potassium chloride (88.15 kg K ha^-1^) | 1. NPK:   N: urea (176 kg N ha^-1^)  P: calcium superphosphate (88.15 kg P ha^-1^)  K: potassium chloride (88.15 kg K ha^-1^)   1. NPKM:   NPK+Rice stalks, bean stalks, cotton seed hulls (100:40:45 compost). Apply the same amount of N as the NPK treatment) |
| CW | Shanxi | 35°12′00″N,  107°40′12″E | 575mm, 9.85℃ | Cumulic Haplustoll | Wheat | 1. Control:   No fertilizer input   1. NK:   N: urea (90 kg N ha^-1^)  K:potassium chloride (90 kg K ha^-1^) | 1. NPK:   N: urea (90 kg N ha^-1^)  P: calcium superphosphate (79 kg P ha^-1^)  K: potassium chloride (90 kg K ha^-1^)   1. NPKM:   NP+75 kg barnyard manure ha^-1^) |
| YT | Sichuan | 31°16′12″N, 105°27′00″E | 812mm, 17.17℃ | Entisol | Maize-  wheat  rotation | 1. Control:   No fertilizer input   1. NK:   N: urea (140 kg N ha^-1^)  K:potassium chloride (36 kg K ha^-1^) | 1. NPK:   N: urea (140 kg N ha^-1^)  P: calcium superphosphate (90 kg P ha^-1^)  K: potassium chloride (36 kg K ha^-1^)   1. NPKM:   NPK+12 t commercial manure ha^-1^) |
| QY | Hunan | 26°45′00″N, 111°52′48″E | 1309mm, 18.78℃ | Hapludult | Maize-  wheat  rotation | 1. Control:   No fertilizer input   1. NK:   N: urea (150 kg N ha^-1^)  K:potassium chloride (50 kg K ha^-1^) | 1. NPK:   N: urea (150 kg N ha^-1^)  P: calcium superphosphate (50 kg P ha^-1^)  K: potassium chloride (60 kg K ha^-1^)   1. NPKM:   NPK+2.25 t commercial manure ha^-1^) |
| FK | Xinjiang | 44°16'59″N, 87°55'1"E | 160mm,  7.03 ℃ | Entisol | Wheat | 1. Control:   No fertilizer input   1. NK:   N: urea (150 kg N ha^-1^)  K: potassium chloride (50 kg K ha^-1^) | 1. NPK:   N: urea (150 kg N ha^-1^)  P: calcium superphosphate (26.5 kg P ha^-1^)  K: potassium chloride (50 kg K ha^-1^)   1. NPKM:   NPK+60 t commercial manure ha^-1^) |

FQ, Fengqiu; CW, Changwu; YT, Yangting; QY, Qiyang; FK, Fukang. MP, average annual precipitation; MT, average annual temperature

**Table S2.** Soil chemical properties of low and high resource availability environments at each sampling site.

| **Sites** | **Resources availability** | **pH** | **OM** | **SOC** | **TN** | **TP** | **TK** | **NO^3-^** | **NH^4+^** | **AP** | **AK** | **C:N** | **C:P** |
| --- | --- | --- | --- | --- | --- | --- | --- | --- | --- | --- | --- | --- | --- |
|  |  |  | **(%)** | **(g kg^-1^)** | **(g kg^-1^)** | **(g kg^-1^)** | **(g kg^-1^)** | **(mg kg^-1^)** | **(mg kg^-1^)** | **(mg kg^-1^)** | **(mg kg^-1^)** |  |  |
| FQ | Low | 8.407 (0.087) | 0.727 (0.011) | 4.215 (0.158) | 0.505 (0.043) | 0.511 (0.015) | 19.655 (0.543) | 38.025 (13.534) | 1.057 (0.367) | 2.12 (0.343) | 240.53 (77.438) | 8.382 (0.648) | 8.252 (0.333) |
|  | High | 8.055 (0.015) | 1.368 (0.117) | 7.937 (1.669) | 0.908 (0.18) | 0.853 (0.034) | 19.625 (0.296) | 45.752 (8.351) | 0.4 (0.044) | 17.64 (0.853) | 224.942 (7.037) | 8.728 (0.23) | 9.348 (2.175) |
| CW | Low | 8.188 (0.078) | 1.287 (0.027) | 7.463 (0.381) | 0.975 (0.07) | 0.755 (0.054) | 20.81 (0.573) | 29.768 (12.455) | 0.558 (0.087) | 4.713 (0.273) | 179.977 (7.073) | 7.676 (0.429) | 9.932 (0.849) |
|  | High | 8.347 (0.144) | 1.827 (0.146) | 10.596 (2.08) | 1.262 (0.232) | 1.118 (0.075) | 20.879 (0.341) | 19.697 (5.426) | 0.83 (0.083) | 35.223 (5.812) | 437.995 (75.787) | 8.38 (0.237) | 9.574 (2.285) |
| YT | Low | 8.378 (0.033) | 0.947 (0.036) | 5.491 (0.509) | 0.749 (0.06) | 0.588 (0.029) | 21.157 (0.261) | 5.633 (0.587) | 1.385 (0.14) | 2.562 (0.317) | 109.637 (5.248) | 7.327 (0.223) | 9.344 (0.671) |
|  | High | 8.242 (0.04) | 1.363 (0.085) | 7.908 (1.212) | 1.02 (0.133) | 0.995 (0.193) | 21.197 (0.78) | 14.96 (0.468) | 1.222 (0.325) | 15.538 (1.403) | 156.61 (18.27) | 7.743 (0.312) | 8.448 (3.422) |
| QY | Low | 4.75 (0.317) | 1.337 (0.022) | 7.753 (0.313) | 0.943 (0.042) | 0.458 (0.01) | 14.319 (0.303) | 7.075 (2.028) | 2.723 (0.568) | 2.188 (0.305) | 139.805 (45.983) | 8.231 (0.406) | 16.925 (0.435) |
|  | High | 5.123 (0.389) | 2.492 (0.174) | 14.453 (2.47) | 1.538 (0.256) | 1.775 (0.502) | 13.963 (1.226) | 8.483 (2.404) | 7.08 (1.492) | 26.822 (1.119) | 355.853 (32.45) | 9.394 (0.267) | 8.381 (1.028) |
| FK | Low | 8.995 (0.044) | 0.948 (0.052) | 5.501 (0.739) | 0.597 (0.068) | 0.983 (0.03) | 21.27 (0.435) | 3.603 (0.81) | 0.808 (0.097) | 3.35 (0.466) | 287.46 (6.485) | 9.208 (0.325) | 5.596 (0.718) |
|  | High | 8.83 (0.031) | 1.337 (0.071) | 7.753 (1.007) | 0.785 (0.104) | 1.234 (0.144) | 20.471 (0.325) | 5.513 (0.295) | 0.81 (0.099) | 26.345 (6.414) | 236.023 (10.777) | 9.89 (0.354) | 6.285 (0.405) |

Data are means of 6 replicates with standard deviation in the parentheses. pH, pH value; OM, organic matter; SOC, soil organic carbon; TN, total nitrogen; TP, total phosphorus; TK, total potassium; AP, available phosphorus; AK, available potassium; NO^3-^, nitrate nitrogen; NH^4+^, ammonium nitrogen; C:N, Ratio of SOC to TN; C:P, Ratio of SOC to TP. FQ, Fengqiu; CW, Changwu; YT, Yangting; QY, Qiyang; FK, Fukang.

**Table S3.** Indicator weight of each experimental site and calculation formula of indicator score.

| **Site** | **Indicator** | **OM** | **pH** | **AP** | **AK** |
| --- | --- | --- | --- | --- | --- |
| FQ | Weight | 0.4094488 | 0.1417323 | 0.2204724 | 0.1889764 |
|  | a | 0.005431 | 0.20972 | 0.000102 | 0.00001 |
|  | c | 18.219012 | 6.77605 | 79.043468 | 277.30496 |
|  | L | 0 | 0 | 0 | 0 |
|  | U | 18.2 | 9.5 | 79 | 277 |
| CW | Weight | 0.3747178 | 0.131678 | 0.2701279 | 0.2234763 |
|  | a | 0.006107 | 0.225097 | 0.001821 | 0.000026 |
|  | c | 27.680348 | 6.685037 | 38.076968 | 293.75838 |
|  | L | 0 | 0.4 | 0 | 0 |
|  | U | 27.7 | 13 | 38.1 | 294 |
| YT | Weight | 0.3678251 | 0.204998 | 0.2210074 | 0.2061695 |
|  | a | 0.001725 | 0.19248 | 0.000253 | 0.000049 |
|  | c | 37.52 | 6.85455 | 63.712849 | 205.2539 |
|  | L | 1 | 3 | 0.1 | 5 |
|  | U | 37.5 | 9 | 64 | 205 |
| QY | Weight | 0.3034292 | 0.2493938 | 0.2071354 | 0.2400416 |
|  | a | 0.002163 | 0.256941 | 0.0038 | 0.000068 |
|  | c | 38 | 6.7 | 40 | 205 |
|  | L | 6 | 4 | 5 | 30 |
|  | U | 38 | 9.5 | 40 | 205 |
| FK | Weight | 0.3988858 | 0 | 0.3314763 | 0.2696379 |
|  | a | 0.001245 | 0.256941 | 0.001293 | 0.000021 |
|  | c | 39.976682 | 6.7 | 41.023703 | 315.8129 |
|  | L | 2 | 4 | 2 | 20 |
|  | U | 39 | 9.5 | 40 | 315 |
| Function type | | Upper function | Peak function | Upper function | Upper function |
| Model of indicator score function | | S=1/(1+a(u-c)^2^) | S=1/(1+a(u-c)^2^) | S=1/(1+a(u-c)^2^) | S=1/(1+a(u-c)^2^) |

S is the indicator score; a is the coefficient; u is the measured value; c is the standard indicator; L is the lower limit and U is the upper limit. When the function type is the upper and u is less than or equal to the lower limit, S is 0; when u is greater than or equal to the upper limit, S is 1; when the function type is peak, u is less than or equal to the lower limit or u is greater than or equal to the upper limit, S is 0.

**Table S4.** Primer information for bacteria, fungi, protists, and nematodes.

| **Belowground organism** | **Region** | **Direction** | **Primer name** | **Primer sequence** | **Amplicon size** | **Reference** |
| --- | --- | --- | --- | --- | --- | --- |
| Bacteria | V4 region of 16s rRNA | Forward | 515F | 5'-GTGCCAGCMGCCGCGGTAA-3' | 253 bp | Caporaso, J.G. et al. [23] |
|  |  | Reverse | 806R | 5'-GGACTACHVGGGTWTCTAAT-3' |  |  |
| Fungi | ITS region | Forward | ITS7F | 5'-GTGARTCATCGARTCTTTG-3' | 500 bp | Ihrmark, K. et al. [24] |
|  |  | Reverse | ITS4R | 5'-TCCTCCGCTTATTGATATGC-3' |  |  |
| Protist | V4 region of 18S rRNA | Forward | S615FC | 5'-GTTAAAAAGCTCGTAGTTG-3' | 350 bp | Fiore-Donno, A.M. et al. [25] |
|  |  |  | S615FP | 5'-GTTAAAARGCTCGTAGTCG-3' |  |  |
|  |  | Reverse | S963R | 5'-CAACTTTCGTTCTTGATYAAA-3' |  |  |
|  |  |  | S947RP | 5'-AAGARGACATCCTTGGTG-3' |  |  |
|  |  |  | S947RV | 5'-AAGAAGATATCCTTGGTG-3' |  |  |
| Nematodes | V4 region of 18S rRNA | Forward | 3ndf | 5'-GGCAAGTCTGGTGCCAG-3' | 570 bp | Geisen, S. et al. [10] |
|  |  | Reverse | 1132rmod | 5'-TCCGTCAATTYCTTTAAGT-3' |  |  |

**Table S5.** Classification of potential species interaction types among belowground organisms (bacteria, fungi, protists, and nematodes).

| **Association types** | **Potential species interactions** |
| --- | --- |
| Positive within trophic | Bacteria↔Bacteria (+) |
|  | Bacteria↔Fungi (+) |
|  | Fungi↔Fungi (+) |
|  | Protists ↔ Protists (+) |
|  | Nematode↔Nematode (+) |
| Negative within trophic | Bacteria↔Bacteria (-) |
|  | Bacteria↔Fungi (-) |
|  | Fungi↔Fungi (-) |
|  | Protists ↔ Protists (-) |
|  | Nematode↔Nematode (-) |
| Positive cross-trophic | Bacteria↔Nematode (+) |
|  | Bacteria↔ Protists (+) |
|  | Fungi↔Protists (+) |
|  | Fungi↔Nematode (+) |
|  | Protists ↔Nematode (+) |
| Negative cross-trophic | Bacteria↔Nematode (-) |
|  | Bacteria↔ Protists (-) |
|  | Fungi↔ Protists (-) |
|  | Fungi↔Nematode (-) |
|  | Protists ↔Nematode (-) |

Association between species can be classified by a combination of interaction coefficient signs +, −, or 0 [26]. Therefore, we use + and - to represent positive and negative associations, respectively. Double arrows indicate associations between two species.

**Table S6.** Fitting index of Partial Least Squares Structural Equation Modeling (PLS-SEM).

| **Model fitting index** | **Resources availability** | **Cronbach's Alpha** | **Composite Reliability** | **Average Variance Extracted (AVE)** | | **Communality** | **R^2^** | **GoodFit** |
| --- | --- | --- | --- | --- | --- | --- | --- | --- |
| **Soil properties** | Low | 0.708 | 0.883 | | 0.806 | 0.645 | - | 0.719 |
|  | High | 0.752 | 0.741 | | 0.773 | 0.557 | - |  |
| **Climate factors** | Low | 0.941 | 0.97 | | 0.942 | 0.654 | - |  |
|  | High | 0.941 | 0.971 | | 0.943 | 0.662 | - |  |
| **Within trophic association** | Low | 0.668 | 0.998 | | 0.998 | 0.787 | 0.763 |  |
|  | High | 0.751 | 0.667 | | 0.993 | 0.775 | 0.878 |  |
| **Cross-trophic association** | Low | 0.861 | 0.933 | | 0.875 | 0.617 | 0.584 |  |
|  | High | 0.739 | 0.841 | | 0.727 | 0.625 | 0.696 |  |
| **Biodiversity** | Low | 1 | 1 | | 1 | 1 | 0.587 |  |
|  | High | 1 | 1 | | 1 | 1 | 0.548 |  |
| **Functional trait stability** | Low | 1 | 1 | | 1 | 1 | 0.665 |  |
|  | High | 1 | 1 | | 1 | 1 | 0.607 |  |

**Table S7.** Properties of ecological co-occurrence networks of soil cross-biome.

| **Sites** | **Resources availability** | **Modularity** | **Nodes** | **Total links** | | **Within trophic links** | | **Cross-trophic links** | |
| --- | --- | --- | --- | --- | --- | --- | --- | --- | --- |
|  |  |  |  | **Positive** | **Negative** | **Positive** | **Negative** | **Positive** | **Negative** |
| Integrate | Low | 0.562 | 4609 | 602202 | 46273 | 595990 | 42950 | 6212 | 3323 |
|  | High | 0.598 | 4522 | 622612 | 70504 | 605004 | 66180 | 17608 | 4324 |
| FQ | Low | 0.473 | 3155 | 9689 | 1379 | 9537 | 1335 | 152 | 44 |
|  | High | 0.811 | 3305 | 6793 | 1571 | 6650 | 1521 | 143 | 50 |
| CW | Low | 0.418 | 3519 | 9926 | 1540 | 9724 | 1505 | 202 | 35 |
|  | High | 0.477 | 3451 | 10299 | 1713 | 9950 | 1642 | 349 | 71 |
| YT | Low | 0.346 | 4115 | 16358 | 1682 | 16032 | 1642 | 326 | 40 |
|  | High | 0.382 | 4117 | 17370 | 2585 | 16887 | 2500 | 483 | 85 |
| QY | Low | 9.598 | 891 | 7510 | 5944 | 6885 | 5301 | 625 | 643 |
|  | High | 12.66 | 1000 | 9063 | 8064 | 8277 | 7362 | 786 | 702 |
| FK | Low | 0.827 | 2461 | 5323 | 2372 | 5083 | 2247 | 240 | 125 |
|  | High | 0.684 | 2483 | 4853 | 1841 | 4618 | 1729 | 235 | 112 |

FQ, Fengqiu; CW, Changwu; YT, Yangting; QY, Qiyang; FK, Fukang.

**References:**

1. Kader M, Lamb DT, Correll R, Megharaj M, Naidu R. Pore-water chemistry explains zinc phytotoxicity in soil. Ecotoxicol Environ Saf. 2015;122:252–9.

2. Nelson DW. Total Carbon , Organic Carbon , and Organic Matter. 1996;961–1010.

3. Bremner JM. Determination of nitrogen in soil by the Kjeldahl method. J Agric Sci. 1960;55:11–33.

4. O’Halloran IP, Cade-Menun BJ. Total and organic phosphorus. In: Carter MR, Gregorich EG, editors. editors Soil sampling and methods of analysis (2nd ed) part III–soil chemical analyses. Boca Raton: CRC Press; 2007. p. 267–71

5. Kanehiro Y SG. Fusion with sodium carbonate for total elemental analysis. In: Black CA, editor. Methods of soil analysis, part 2–agronomy 9. Madison: America Society of Agronomy, Inc; 1965. p. 952–8.

6. Lu K. Analytical methods of soil and agricultural chemistry. China. Soil Ecol. Beijing, China: Agricultural Science and Technology; 1999.

7. Chen Y, Bonkowski M, Shen Y, Griffiths BS, Jiang Y, Wang X, et al. Root ethylene mediates rhizosphere microbial community reconstruction when chemically detecting cyanide produced by neighbouring plants. Microbiome. 2020;8.

8. Sun R, Chen Y, Han W, Dong W, Zhang Y, Hu C, et al. Different contribution of species sorting and exogenous species immigration from manure to soil fungal diversity and community assemblage under long-term fertilization. Soil Biol Biochem. Elsevier Ltd; 2020;151:108049.

9. Fiore-Donno AM, Rixen C, Rippin M, Glaser K, Samolov E, Karsten U, et al. New barcoded primers for efficient retrieval of cercozoan sequences in high-throughput environmental diversity surveys, with emphasis on worldwide biological soil crusts. bioRxiv. 2017;

10. Geisen S, Snoek LB, ten Hooven FC, Duyts H, Kostenko O, Bloem J, et al. Integrating quantitative morphological and qualitative molecular methods to analyse soil nematode community responses to plant range expansion. Methods Ecol Evol. 2018;9:1366–78.

11. Bolger AM, Lohse M, Usadel B. Trimmomatic: A flexible trimmer for Illumina sequence data. Bioinformatics. 2014;30:2114–20.

12. Aronesty E. Comparison of Sequencing Utility Programs. Open Bioinforma J. 2013;7:1–8.

13. Martin M. Cutadapt removes adapter sequences from high-throughput sequencing reads. Embnet J. 2011;17:10–2.

14. Rognes T, Flouri T, Nichols B, Quince C, Mahé F. VSEARCH: A versatile open source tool for metagenomics. PeerJ. 2016;2016:1–22.

15. Wang Q, Garrity GM, Tiedje JM, Cole JR. Naive Bayesian classifier for rapid assignment of rRNA\nsequences into the new bacterial taxonomy. Appl Environ Microbiol. 2007;73:5261–7.

16. Yuan MM, Guo X, Wu L, Zhang Y, Xiao N, Ning D, et al. Climate warming enhances microbial network complexity and stability. Nat Clim Chang. 2021.

17. Guo X, Zhou X, Hale L, Yuan M, Ning D, Feng J, et al. Climate warming accelerates temporal scaling of grassland soil microbial biodiversity. Nat Ecol Evol. Springer US; 2019;3:612–9.

18. Tu Q, Yu H, He Z, Deng Y, Wu L, Van Nostrand JD, et al. GeoChip 4: A functional gene-array-based high-throughput environmental technology for microbial community analysis. Mol Ecol Resour. 2014;14:914–28.

19. Shi Z, Yin H, Van Nostrand JD, Voordeckers JW, Tu Q, Deng Y, et al. Functional Gene Array-Based Ultrasensitive and Quantitative Detection of Microbial Populations in Complex Communities. mSystems. 2019;4.

20. Xue K, Yuan MM, Shi ZJ, Qin Y, Deng Y, Cheng L, et al. Tundra soil carbon is vulnerable to rapid microbial decomposition under climate warming. Nat Clim Chang. 2016;6:595–600.

21. Li D, Ni H, Jiao S, Lu Y, Zhou J, Sun B, et al. Coexistence patterns of soil methanogens are closely tied to methane generation and community assembly in rice paddies. Microbiome. Microbiome; 2021;9:1–14.

22. Cheng L, Zhang N, Yuan M, Xiao J, Qin Y, Deng Y, et al. Warming enhances old organic carbon decomposition through altering functional microbial communities. ISME J. Nature Publishing Group; 2017;11:1825–35.

23. Caporaso JG, Lauber CL, Walters WA, Berg-Lyons D, Lozupone CA, Turnbaugh PJ, et al. Global patterns of 16S rRNA diversity at a depth of millions of sequences per sample. Proc Natl Acad Sci U S A. 2011;108:4516–22.

24. Ihrmark K, Bödeker ITM, Cruz-Martinez K, Friberg H, Kubartova A, Schenck J, et al. New primers to amplify the fungal ITS2 region - evaluation by 454-sequencing of artificial and natural communities. FEMS Microbiol Ecol. 2012;82:666–77.

25. Fiore-Donno AM, Rixen C, Rippin M, Glaser K, Samolov E, Karsten U, et al. New barcoded primers for efficient retrieval of cercozoan sequences in high-throughput environmental diversity surveys, with emphasis on worldwide biological soil crusts. Mol Ecol Resour. 2018;18:229–39.

26. Odum EP, Valiela I. Basic Ecology. Q Rev Biol. 1983;34.
